# GPNMB is a biomarker for lysosomal dysfunction and is secreted via LRRK2-modulated lysosomal exocytosis

**DOI:** 10.1126/sciadv.adv1434

**Published:** 2025-12-17

**Authors:** Erin C. Bogacki, Gustavo Morrone Parfitt, Adriana Cunha, George Longmore, Selina Wray, Patrick A. Lewis, Susanne Herbst

**Affiliations:** ^1^ Department of Comparative Biomedical Sciences, Royal Veterinary College, Camden, NW1 0TU, UK.; ^2^ Department of Neurodegenerative Disease, UCL Queen Square Institute of Neurology, London, WC1N 3BG, UK.; ^3^ Aligning Science Across Parkinson’s Research Network, Chevy Chase, MD 20815, USA.

## Abstract

Genome-wide association studies have identified *Glycoprotein Nmb* (*GPNMB*) as a risk factor for Parkinson’s disease. The risk allele increases *GPNMB* transcription and GPNMB protein levels in the CSF highlighting GPMNB as a potential biomarker for Parkinson’s disease. However, a lack of knowledge of GPNMB’s function and mechanism of secretion has hindered an interpretation of secreted GPNMB levels. In this study, we assessed the mechanism of GPNMB secretion by macrophages, the primary cell type expressing GPNMB in the brain. We show that GPNMB is secreted in response to lysosomal stress via lysosomal exocytosis and highlight the Parkinson’s disease risk factor LRRK2 as a strong modulator of GPNMB secretion.

## Teaser

The secretion mechanism of the Parkinson’s disease risk factor GPNMB highlights lysosomal dysfunction as a disease mechanism.

## INTRODUCTION

In recent years, our understanding of the genetic basis of Parkinson’s disease (PD) has expanded with genome-wide association studies now highlighting over 90 gene loci which contribute to the development of the disease (*1*). This expansion and refinement of the genetic architecture for PD have resulted in the new challenge of translating the genetic findings into therapeutic targets and biomarkers for this disorder.

One of the genes identified by genome-wide association is *Glycoprotein Nmb* (*GPNMB*), with the risk allele resulting in a 10% increased lifetime risk of developing PD. GPNMB, the protein product of *GPNMB,* is a transmembrane protein with a secreted extracellular domain. The *GPNMB* risk polymorphism results in increased *GPNMB* expression (*2*, *3*), and increased levels of the GPNMB secreted extracellular domain in the cerebrospinal fluid (CSF) (*4*)—suggesting a role for increased secreted GPNMB levels in the pathogenesis of PD. In contrast, *GPNMB* loss-of-function mutations result in autosomal-recessive amyloidosis cutis dyschromica (ACD), which manifests in abnormal skin pigmentation (*5*, *6*). GPNMB is homologous to melanosome amylogenic protein PMEL and therefore is thought to play a role in melanosome biogenesis, which might underlie its role in the etiology of ACD (*7*). In the context of PD, recent studies suggest that GPNMB can act as a receptor for α-synuclein uptake in neurons (*3*). However, GPNMB knockout (KO) failed to prevent the spread of α-synuclein or loss of dopaminergic neurons in several murine PD models (*4*). Therefore, the pathogenic mechanism of either intracellular or secreted GPNMB in PD remains elusive. GPNMB is highly expressed in microglia and has been described as a marker for proinflammatory microglia in PD (*4*, *8*).

*GPNMB* is under the transcriptional control of transcription factor EB (TFEB), a lysosomal stress–responsive transcription factor (*9*). This has led to GPNMB being proposed as a lysosomal protein, aligning with a wider array of PD risk genes that highlight lysosomal dysfunction as a causal factor in PD (*10*). For example, variation in the lysosomal enzyme GBA1 underlies common PD risk in diverse populations (*1*, *11*), and mutations in leucine-rich repeat kinase 2 (LRRK2), which is recruited to stressed lysosomes and has been linked to lysosomal trafficking (*12*–*14*), represent one of the most common inherited forms of PD. However, little is known about the physiological events that induce GPNMB secretion, or whether GPNMB function aligns with other lysosomal contributors to PD. These gaps in our knowledge hinder the interpretation of GPNMB as a secreted biomarker or the evaluation of GPNMB as a therapeutic target in PD.

This study investigated pathways modulating GPNMB secretion, revealing that dysfunctional lysosomes recruit GPNMB, leading to the secretion of the GPNMB extracellular domain by lysosomal exocytosis. Linking *GPNMB* to monogenic forms of Parkinson’s, CSF levels of secreted GPNMB are strongly increased in *LRRK2* mutation carriers, as well as *GBA1* mutation carriers and in idiopathic PD (iPD). These data suggest that secreted GPNMB can be considered a biomarker of lysosomal stress and that this can reflect lysosomal dysfunction in genetic cases of Parkinson’s.

## RESULTS

### Macrophages secrete GPNMB in response to lysosomal stress

**Fig. 1.**
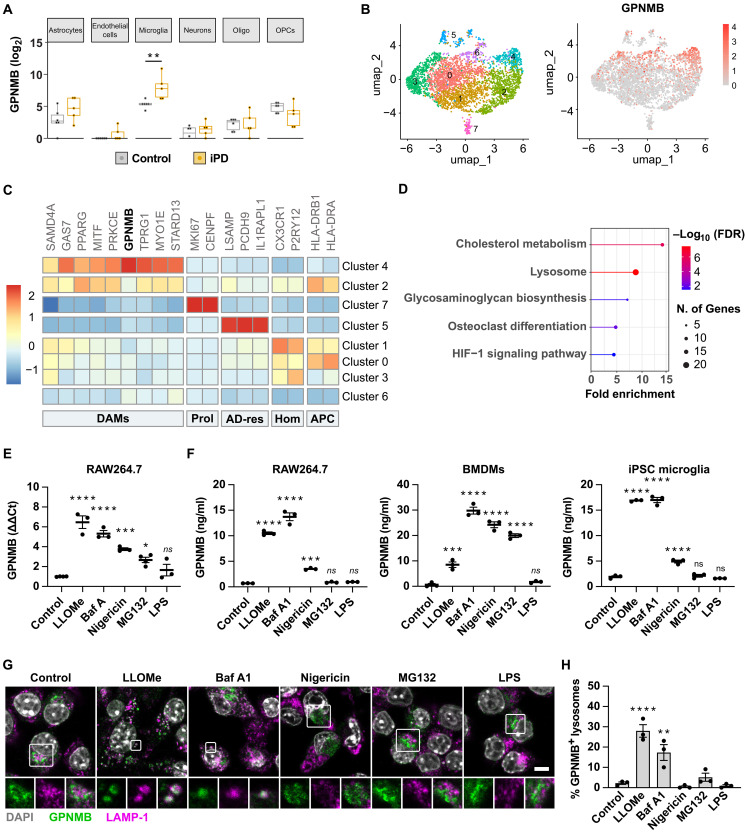
Macrophages secret GPNMB in response to lysosomal stress. (**A**) Average GPNMB expression across different cell types in the midbrain in controls and iPD cases. Analyzed from (*8*). (**B**) Subclustering of microglia from (A) identifies a GPNMB high expression cluster. (**C**) Heatmap of average expression of genes associated with disease-associated microglia (DAMs), proliferating cells (Prol.), AD-resistant microglia (AD-res), homeostatic microglia (Hom.), and antigen-presenting microglia (APC). (**D**) Pathway enrichment analysis of DEGs of GPNMB-expressing DAM cluster. HIF-1, hypoxia-inducible factor 1; FDR, false discovery rate. (**E**) RAW264.7 macrophages were either left untreated or treated with LLOMe (500 μM), bafilomycin A1 (100 nM), nigericin (5 μM), MG132 (5 μM), or LPS (10 ng/ml) for 4 hours, and GPNMB transcription was assessed by qPCR. (**F**) RAW264.7 macrophages, BMDMs, or iPSC-derived microglia were either left untreated or treated with LLOMe (500 μM), bafilomycin A1 (10 nM), nigericin (1 μM), MG132 (5 μM), or LPS (10 ng/ml) overnight. GPNMB secretion was assessed by enzyme-linked immunosorbent assay (ELISA). (**G** and **H**) RAW264.7 macrophages were either left untreated or treated with LLOMe (1 mM for 1 hour), bafilomycin A1 (100 nM for 3 hours), nigericin (5 μM for 1 hour), MG132 (5 μM), or LPS (10 ng/ml for 1 hour), and GPNMB–LAMP-1 colocalization was assessed by confocal microscopy. (G) Mean ± SEM from three independent experiments. (H) Representative images. Scale bar, 5 μm. ns, not significant.

The *GPNMB* PD risk allele results in increased GPNMB CSF concentrations (*4*), and previous reports have highlighted increased GPNMB levels in the plasma of patients with PD (*3*). To identify the source and potential triggers of GPNMB secretion in PD, we reanalyzed a midbrain single nucleus RNA sequencing dataset of age-matched controls and patients with iPD (*8*). *GPNMB* expression was the highest in microglia, followed by astrocytes and oligodendrocyte precursors. However, *GPNMB* expression was only increased in iPD samples in microglia, pointing to microglia as a PD-responsive source of GPNMB (Fig. 1A). As previously reported (*4*, *15*), microglia subclustering identified disease-associated microglia (DAMs) as the main subtype expressing *GPNMB* (Fig. 1, B and C). Pathway enrichment analysis of genes differentially expressed in the DAM cluster highlighted dysregulation of cholesterol metabolism, lysosomal activation, and hypoxic stress as potential microglia stressors (Fig. 1D). Congruent with this, *GPNMB* transcription is regulated by the MiT/TFE family of transcription factors (*9*, *16*), indicating a role for GPNMB in the lysosomal stress response. To test the effect of lysosomal stress on GPNMB secretion, we first confirmed the transcriptional up-regulation of GPNMB in response to various lysosomal stressors, such as the lysosomal damaging agent LLOMe, the lysosomal Vacuolar-type ATPase inhibitor bafilomycin A1, and the K^+^/H^+^ antiporter nigericin in RAW264.7 mouse macrophages. Lysosomal damage and alkalinization resulted in strong up-regulation of *GPNMB* transcription, whereas nigericin had a more modest effect (Fig. 1E). In addition, we observed minor up-regulation of *GPNMB* gene transcription in response to proteotoxic stress induced by inhibition of the proteasome with MG132, but no transcriptional up-regulation after treatment with the Toll-like receptor 4 ligand lipopolysaccharide (LPS; Fig. 1E). Next, we assessed GPNMB secretion in the mouse macrophage cell line RAW264.7, mouse bone marrow–derived macrophages (BMDMs), and induced pluripotent stem cell (iPSC)-derived microglia. In all cases, direct lysosomal stress resulted in GPNMB secretion, whereas the effect of indirect stressors such as MG-132 varied. In contrast, LPS treatment did not result in GPNMB secretion. Overall, lysosomal damage induced by LLOMe and lysosomal alkalinization with the V-ATPase inhibitor bafilomycin A1 resulted in reproducible secretion of GPNMB across the different cell types tested (Fig. 1F). To test whether GPNMB secretion is merely a consequence of transcriptional up-regulation or an active process, we treated RAW264.7 macrophages with cycloheximide to inhibit protein synthesis or introduced tetracycline-inducible *GPNMB* expression into GPNMB KO RAW264.7 macrophages. As expected, cycloheximide treatment reduced the total amount of GPNMB secreted, but we still observed induction of GPNMB secretion in the presence of cycloheximide in response to bafilomycin A1 treatment (fig. S1, A and B). Similarly, when *GPNMB* expression was transcriptionally uncoupled from the lysosomal stress response by placing it under a tetracycline-inducible promoter, we only observed GPNMB secretion in response to bafilomycin A1 treatment despite an increase in GPNMB protein levels (fig. S1, C and D). These data indicate that GPNMB transcriptional up-regulation and its secretion can be uncoupled.

To gain an insight into the mechanisms of GPNMB secretion, we assessed GPNMB subcellular localization in response to the same lysosomal and cellular stressors. GPNMB expression was found to be heterogeneous across cells, most likely reflecting differences in MiT/TFE transcriptional activity across cells (Fig. 1G). When expressed, GPNMB was predominantly localized to the perinuclear region (Fig. 1G). We were unable to identify the steady-state localization of GPNMB at endogenous expression levels; however, overexpression of an enhanced green fluorescent protein (EGFP)–tagged GPNMB expression construct in human embryonic kidney (HEK) 293 cells (which do not express detectable GPNMB at an endogenous level) indicated that GPNMB localizes to a perinuclear endosomal compartment (fig. S2). The application of lysosomal stress, however, resulted in the relocalization of endogenous GPNMB to LAMP-1–positive lysosomes, indicating that GPNMB secretion is linked to lysosomal recruitment (Fig. 1, G and H).

### GPNMB lysosomal recruitment is a prerequisite for its secretion

**Fig. 2.**
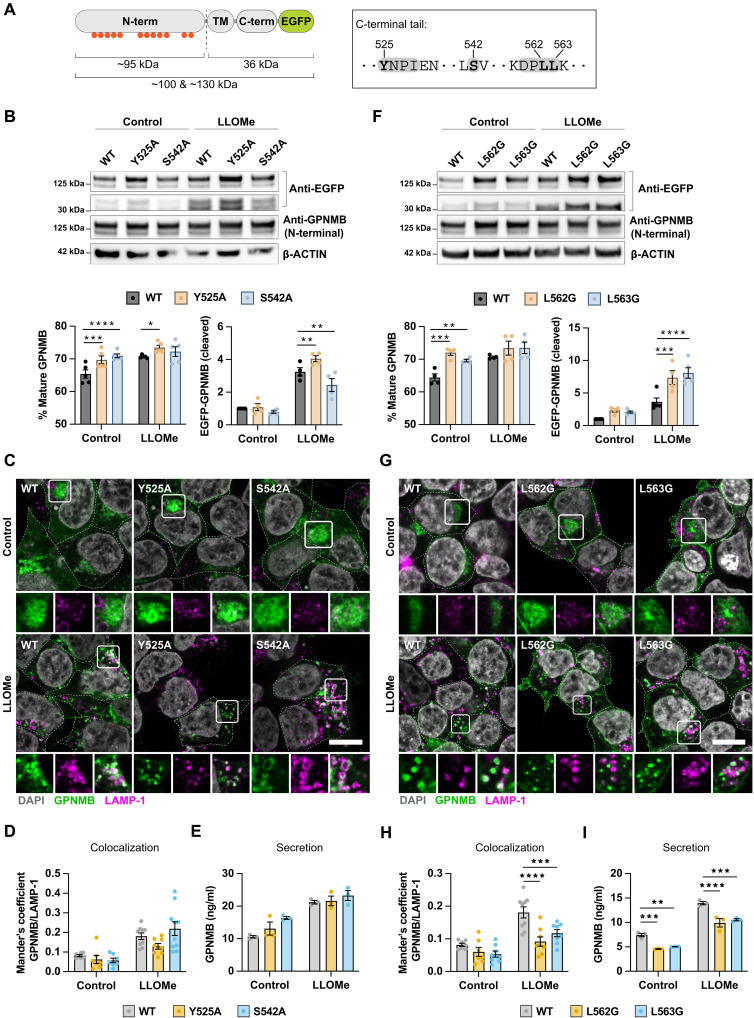
GPNMB lysosomal recruitment is a prerequisite for secretion. (**A**) Overview of the GPNMB overexpression construct with the glycosylation sites marked by red circles and the cleavage site marked by a dashed line, highlighting the sizes of the GPNMB fragments that can be detected using an anti-GFP and an anti-GPNMB antibody. The potential lysosomal recruitment motifs in the C-terminal tail of GPNMB include an HemITAM (Y525), a phosphorylated serine (S542), and a di-Leucine motif (L562 and L563). (**B** to **E**) HEK293 cells were transfected with GPNMB-EGFP WT or containing a Y525A or S542A point mutation. (B) GPNMB processing was assessed by Western blotting for the C-terminal EGFP-tag and with an N-terminal targeting anti-GPNMB antibody. Western blots were quantified using ImageJ. Graphs show mean ± SEM, *n* = 4. (C) GPNMB localization was imaged by confocal microscopy. Scale bar, 10 μm. (D) The fraction of GPNMB overlapping with LAMP-1 was calculated by Mander’s coefficient using the JaCOP plugin in ImageJ. Graphs show mean ± SEM of *n* = 3 with two to four technical replicates each. (E) GPNMB secretion was measured by ELISA after overnight treatment with LLOMe (500 μM). Graphs show mean ± SEM, *n* = 3. (**F** to **I**) HEK293 cells were transfected with GPNMB-EGFP WT or containing a L562G or L563G point mutation. (F) GPNMB processing was assessed by Western blotting for the C-terminal EGFP tag and with an N-terminal targeting anti-GPNMB antibody. WBs were quantified using ImageJ. Graphs show mean ± SEM, *n* = 4. (G) GPNMB localization was imaged by confocal microscopy. Scale bar, 10 μm. (H) The fraction of GPNMB overlapping with LAMP-1 was calculated by Mander’s coefficient using the JaCOP plugin in ImageJ. Graphs show mean ± SEM of *n* = 3 with two to four technical replicates each. (I) GPNMB secretion was measured by ELISA after overnight treatment with LLOMe (500 μM). Graphs show mean ± SEM, *n* = 3.

To test whether GPNMB secretion is dependent on lysosomal recruitment, we mutated motifs within the GPNMB cytosolic tail which are predicted to aid endolysosomal sorting. S542 is the most robust phospho signal reported for GPNMB on Phospho Site Plus (*17*) and forms part of a putative export signal for sorting into clathrin-coated vesicles from the trans-Golgi (*18*). Y525 forms part of a hypothetical HemITAM motif required for AP-4–mediated clathrin-independent sorting, and L562/L563 form the core of a di-leucine motif, a common lysosomal sorting motif (Fig. 2A) (*19*). To understand the impact of the described motifs on GPNMB lysosomal sorting, individual amino acids were mutated, and GPNMB processing, trafficking, and secretion were assessed in a HEK293 cell overexpression system. In this system, we were able to detect the glycosylated precursor and mature form of GPNMB using an anti-GPNMB antibody, which targets the N-terminal part of GPNMB, and a ~35-kDa fragment that represents the cleaved C-terminal tail when using an anti-GFP antibody.

Mutating S542 to A or mutating the proposed HemITAM affected the ratio of mature to precursor protein and altered the amount of the N-terminal cleavage product (Fig. 2B). However, these mutations had no obvious effect on GPNMB localization and secretion before or after LLOMe stimulation (Fig. 2, C to E).

Similar to the HemITAM and S542A mutants, the L562G and L563G mutants showed a higher ratio of mature to precursor form and markedly increased the presence of the C-terminal cleavage product at steady state and after LLOMe stimulation (Fig. 2F). Moreover, the C-terminal di-leucine motif was required for GPNMB lysosomal localization. Both the L562G and L563G mutants displayed reduced localization to LAMP-1–positive vesicles and instead mislocalized to the plasma membrane in response to LLOMe treatment, indicating that transport of GPNMB to lysosomes is dependent on its di-leucine motif (Fig. 2, G and H). In addition, the secretion of the L562G and L563G mutants was reduced, suggesting that GPNMB lysosomal recruitment is a prerequisite for GPNMB secretion (Fig. 2I). These results also indicate that lysosomes might contribute to GPNMB turnover, especially of the C-terminal tail.

### GPNMB is secreted via lysosomal exocytosis

**Fig. 3.**
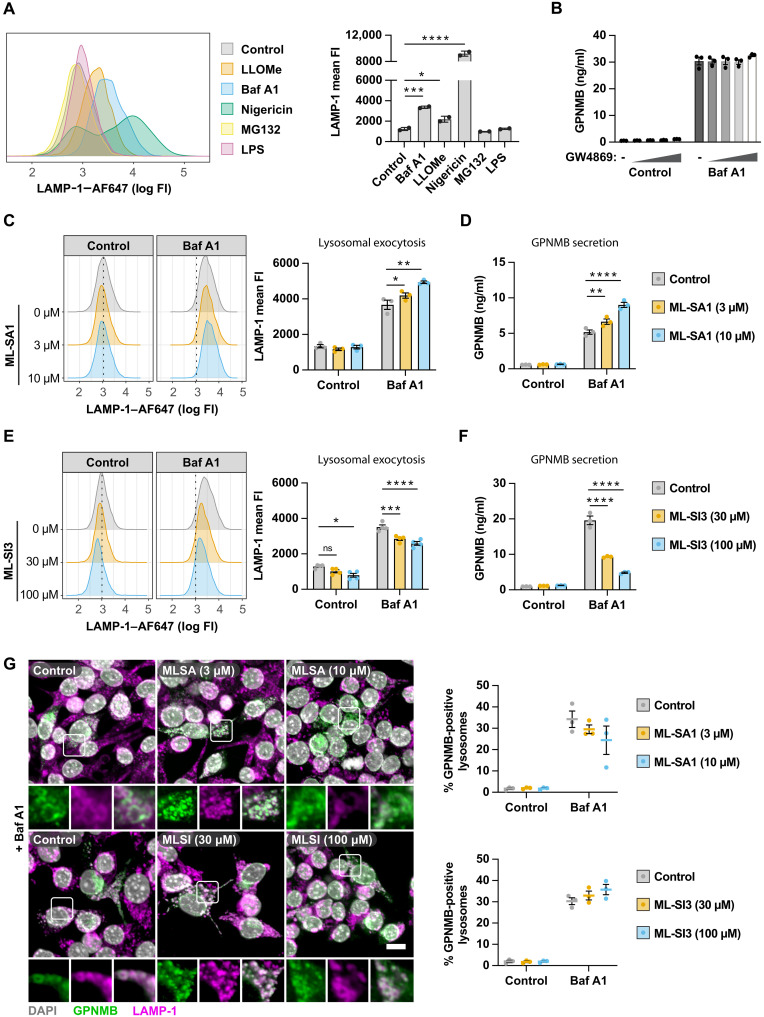
GPNMB is secreted via lysosomal exocytosis. (**A**) Lysosomal exocytosis was estimated by measuring LAMP-1 on the cell surface by flow cytometry. RAW264.7 macrophages were either left untreated or treated with LLOMe (500 μM), bafilomycin A1 (100 nM), nigericin (5 μM), MG132 (5 μM), or LPS (10 ng/ml) for 4 hours. Graphs show mean fluorescence intensity (FI) ± SEM, *n* = 2. (**B**) RAW264.7 macrophages were treated with increasing concentrations of GW4869 (0.3 to 1 to 3 to 10 μM) and stimulated with bafilomycin A1 (10 nM) overnight. GPNMB secretion was measured by ELISA. Mean ± SEM, *n* = 3. (**C**) RAW264.7 macrophages were treated with ML-SA1 and costimulated with bafilomycin A1 (100 nM) for 4 hours. Lysosomal exocytosis was measured by quantifying the cell surface abundance of LAMP-1 by fluorescence-activated cell sorting (FACS). Mean ± SEM, *n* = 3. (**D**) RAW264.7 macrophages were treated with ML-SA1 and costimulated with bafilomycin A1 (10 nM) overnight. GPNMB secretion was measured by ELISA. Mean ± SEM, *n* = 3. (**E**) RAW264.7 macrophages were treated with ML-SI3 and stimulated with bafilomycin A1 (100 nM) for 4 hours. Lysosomal exocytosis was measured by quantifying the cell surface abundance of LAMP-1 by FACS. Mean ± SEM, *n* = 3. (**F**) RAW264.7 macrophages were treated with ML-SI3 and stimulated with bafilomycin A1 (10 nM) overnight. GPNMB secretion was measured by ELISA. Mean ± SEM, *n* = 3. (**G**) RAW264.7 macrophages were treated with ML-SI3 or MLSI and costimulated with bafilomycin A1 (100 nM) for 4 hours. GPNMB localization to LAMP-1–positive lysosomes was assessed by high-content imaging. Mean ± SEM shown, *n* = 3. Scale bar, 10 μm.

These data point to the GPNMB ectodomain being secreted via the lysosome. Lysosomes secret content by fusing with the plasma membrane which results in the translocation of lysosomal membrane protein to the plasma membrane (*20*). Therefore, measuring the abundance of LAMP-1 on the plasma membrane by flow cytometry can serve as a proxy for lysosomal exocytosis. As predicted, LLOMe, bafilomycin A1, and nigericin treatment resulted in lysosomal exocytosis, whereas MG132 and LPS did not result in lysosomal exocytosis (Fig. 3A). As lysosomal content can be released directly or in vesicles, we inhibited intraluminal vesicle formation using GW4869. GW4869 did not affect GPNMB secretion, indicating that GPNMB is not secreted in extracellular vesicles (Fig. 3B). To determine whether lysosomal exocytosis is required for GPNMB secretion, we modulated the activity of the lysosomal Ca2^+^ efflux channel TRPML1 which acts as a key mediator of lysosomal exocytosis (*21*, *22*). The TRPML1 channel agonist ML-SA1 increased lysosomal exocytosis (Fig. 3C) and increased GPNMB secretion (Fig. 3D). Vice versa, the TRPML1 channel antagonist ML-SI3 decreased lysosomal exocytosis (Fig. 3E) and strongly decreased GPNMB secretion (Fig. 3F). We confirmed that the modulation of lysosomal calcium flux did not directly affect the recruitment of GPNMB to lysosomes (Fig. 3G) leading to the conclusion that GPNMB is secreted via lysosomal exocytosis.

### ADAM10 contributes to GPNMB secretion

**Fig. 4.**
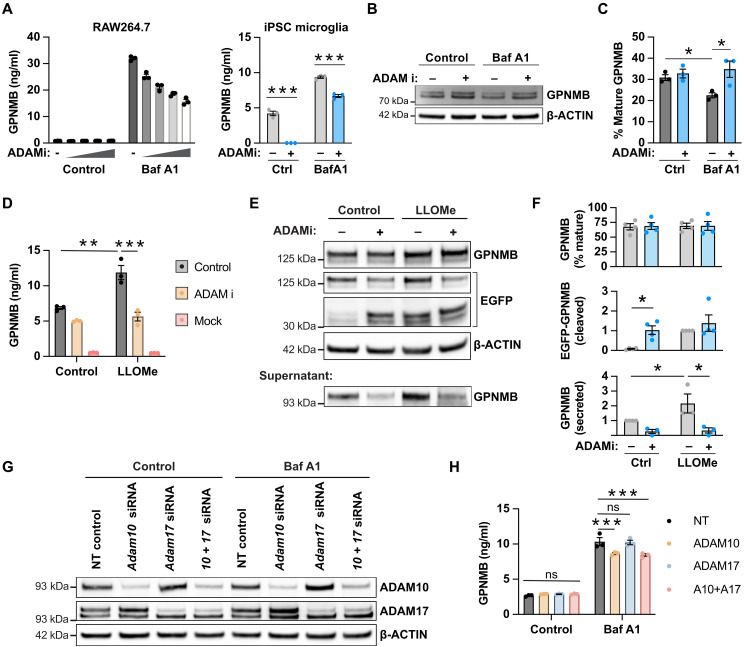
Cleavage by ADAM10 contributes to GPNMB secretion. (**A**) RAW264.7 macrophages and iPSC-derived microglia were treated with the ADAM10/17 inhibitor G280264x (0.3 to 1 to 3 to 10 μM or 3 μM, respectively). GPNMB secretion in response to bafilomycin A1 (10 nM, overnight) was measured by ELISA. Mean ± SEM, *n* = 3. (**B**) RAW264.7 macrophages were pretreated with cycloheximide (5 μg/ml) and the ADAM10/17 inhibitor G280264x (3 μM) where indicated, followed by stimulation with bafilomycin A1 (100 nM) for 4 hours. Endogenous GPNMB protein levels were measured by Western blot. (**C**) Quantification of the mature form of GPNMB as a percentage of total GPNMB from (B). Mean ± SEM, *n* = 3. (**D**) Human GPNMB-EGFP transfected HEK293 cells were pretreated with the ADAM10/17 inhibitor G280264x (3 μM), and GPNMB secretion in response to LLOMe (1 mM, overnight) was measured by ELISA. Mean ± SEM, *n* = 3. Mock indicates the nontransfected control. (**E**) Human GPNMB-EGFP transfected HEK293 cells were pretreated with the ADAM10/17 inhibitor G280264x (3 μM) and stimulated with LLOMe (1 mM). GPNMB protein levels in the cell lysate (1-hour LLOMe treatment) and supernatant (4-hour LLOMe treatment) were measured by Western blot. (**F**) Quantification of the mature form of GPNMB, the EGFP-tagged C-terminal tail, and secreted GPNMB from (B). Mean ± SEM, *n* = 3 to 4. (**G**) RAW264.7 macrophages were treated with 100 nM small interfering RNAs (siRNAs) targeting ADAM10 or ADAM17 or a non-targeting (NT) control and stimulated with bafilomycin A1 (10 nM) overnight. Knockdown efficiency was assessed by Western blot. (**H**) GPNMB secretion was measured by ELISA. Mean ± SEM, *n* = 3.

The secreted N-terminal fragment of GPNMB is thought to be generated by cleavage by α-secretases such as ADAM10 (*23*). To test the requirement for ADAM10 or the closely related ADAM17 for bafilomycin A1 or LLOMe-stimulated GPNMB secretion, we inhibited ADAM10 and ADAM17 using GW 280264X (*24*). In RAW264.7 macrophages and iPSC-derived microglia, ADAM10/17 inhibition reduced but was not able to completely inhibit GPNMB secretion (Fig. 4A). Further, the reduction in mature, fully glycosylated GPNMB due to increased secretion following BafA1 treatment was reversed by ADAM10/17 inhibition (Fig. 4, B and C). To further evaluate processing of GPMNB, we sought to detect the C-terminal intracellular fragment of EGFP-tagged overexpressed GPNMB in HEK293 cells. In agreement with the findings in RAW264.7 macrophages, ADAM10/17 inhibition reduced the secretion of overexpressed GPNMB in HEK293 cells (Fig. 4D). Lysosomal damage induced by treatment with LLOMe increased the amount of the cleaved C-terminal fragment detected with an anti-EGFP antibody, indicating either increased cleavage or impaired degradation as seen in Fig. 2. Unexpectedly, ADAM10/17 inhibition increased the amount of the C-terminal fragment at steady state but did not significantly alter the amount after LLOMe stimulation—supporting a complex interplay between cleavage and degradation (Fig. 4, E and F). However, in the HEK293 overexpression system, we were able to detect secreted GPNMB in the supernatant by Western blotting, confirming that LLOMe treatment increased secretion which was in turn inhibited by the ADAM10/17 inhibitor (Fig. 4, E and F). In line with reduced cleavage and secretion, ADAM10/17 inhibition resulted in an increased presence of GPNMB on lysosomes and the cell surface (fig. S3). To differentiate between cleavage by ADAM10 and ADAM17, we knocked down ADAM10 or ADAM17 in RAW264.7 cells and assessed GPNMB secretion. Despite similar levels of knockdown (Fig. 4G), only ADAM10 knockdown reduced GPNMB secretion (Fig. 4H). However, we noted that despite a high degree of knockdown, the effect on GPNMB secretion was minimal, indicating that either minimal levels of ADAM10 activity are sufficient for cleavage or that alternative ADAM secretases or proteases can cleave GPNMB. We concluded that ADAM10 contributes to GPNMB lysosomal secretion but is unlikely to be the sole protease responsible for cleavage.

### The LRRK2 G2019S pathogenic variant increases GPNMB secretion

**Fig. 5.**
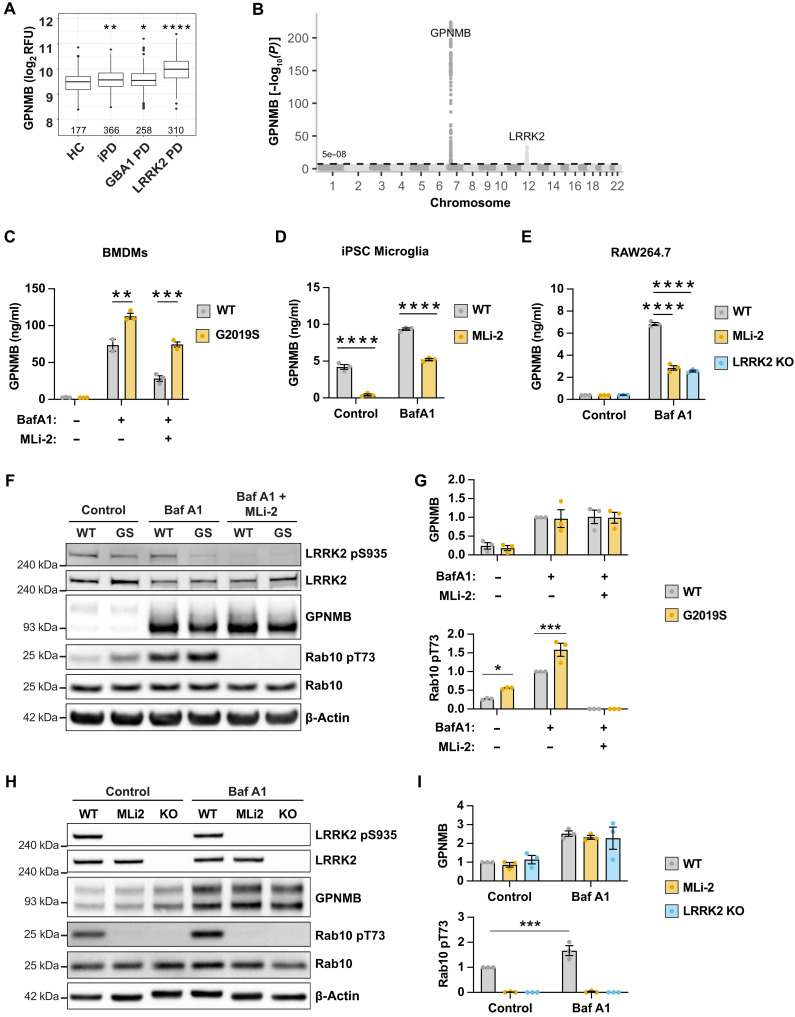
The LRRK2 G2019S mutation increases GPNMB secretion. (**A**) SomaScan data of GPNMB levels (presented in relative fluorescent units, RFU) in the CSF of healthy controls (HC), patients with iPD, patients with GBA1 PD, and patients with LRRK2 G2019S PD indicate higher GPNMB levels in LRRK2 mutation carriers. (**B**) GWAS data correlated to CSF SomaScan data of GPNMB (analyte 8240.207) were downloaded via the Washington University School of Medicine ONTIME browser (*27*). Genetic modifiers of GPNMB CSF levels are highlighted: GPNMB rs199347 (*P* = 6.8 × 10^−225^) and LRRK2 rs76904798 (*P* = 3.9 × 10^−34^). (**C**) WT and LRRK2 G2019S BMDMs were pretreated with MLi-2 (100 nM) and stimulated with bafilomycin A1 (10 nM) overnight. GPNMB levels were measured by ELISA. Mean ± SEM, *n* = 3. (**D**) WT [control and MLi-2 (100 nM) pretreated] and LRRK2 KO RAW264.7 macrophages were stimulated with bafilomycin A1 (10 nM) overnight. GPNMB levels were measured by ELISA. Mean ± SEM, *n* = 3. (**E**) iPSC-derived microglia were pretreated with MLi-2 (100 nM) and stimulated with bafilomycin A1 (100 nM) overnight. GPNMB levels were measured by ELISA. Mean ± SEM, *n* = 3. (**F**) WT and LRRK2 G2019S BMDMs were pretreated with MLi-2 (100 nM) and stimulated with bafilomycin A1 (10 nM) overnight. GPNMB levels and LRRK2 activation were assessed by Western blotting. (**G**) Quantification of total GPNMB protein levels (premature + mature form) and Rab10 T73 phosphorylation from (F). Graph shows mean ± SEM, *n* = 3. (**H**) WT [control and MLi-2 (100 nM) pretreated] and LRRK2 KO RAW264.7 macrophages were stimulated with bafilomycin A1 (10 nM) overnight. GPNMB levels and LRRK2 activation were assessed by Western blotting. (**I**) Quantification of total GPNMB protein levels (premature + mature form) and Rab10 T73 phosphorylation from (H). Graph shows mean ± SEM, *n* = 3.

GPNMB has been proposed as a biomarker for PD, but increased GPNMB serum levels have also been reported in patients with Gaucher’s disease who carry homozygous *GBA1* mutations (*25*), and pharmacological inhibition of glucocerebrosidase increased GPNMB protein levels in the brain of wild-type (WT) mice (*26*). As heterozygous *GBA1* mutations are a prominent risk factor for developing PD, we aimed to address the question of whether defined monogenic forms of PD present with altered GPNMB processing in the central nervous system of patients with PD. We analyzed the Parkinson’s Progression Markers Initiative (PPMI) dataset that includes GPNMB CSF levels of patients with iPD, patients with PD carrying *GBA1* mutations, and patients with PD carrying the *LRRK2 G2019S* mutation. GPNMB levels in patients with iPD were slightly elevated when compared to age-matched, healthy controls as previously reported, and *GBA1* mutation carriers showed elevated GPNMB levels to a similar degree as patients with iPD (Fig. 5A). Unexpectedly, *LRRK2* mutation carriers showed highly elevated GPNMB CSF levels (Fig. 5A) without demonstrating a general release of lysosomal enzymes into the CSF (fig. S4). We further interrogated the Online Neurodegenerative Trait Integrative Multi-Omics Explorer (ONTIME) hosted by Washington University (*27*) for single-nucleotide polymorphisms (SNPs) affecting GPNMB CSF levels across neurodegenerative disease. In agreement with the PPMI dataset, a known PD-risk SNP, rs76904798, which increases LRRK2 levels in microglia (*28*), was the only modifier of GPNMB CSF levels besides a well-described SNP in the *GPNMB* gene (rs199351) (Fig. 5B). Together, these data imply an impact of LRRK2 function on GPNMB biology. The pattern of GPNMB CSF levels paralleled that observed with urinary di-22:6-BMP levels in the same patient cohort (fig. S5A) (*29*). Although LRRK2 activity seems to be driving both the presence of the lysosomal lipid di-22:6-BMP in the urine and the secretion of GPNMB into the CSF, we observed only a weak correlation of GPNMB CSF levels and di-22:6-BMP levels in the urine (fig. S5, B and C), hinting to a difference in triggers of release that require further investigation.

To verify that LRRK2 activity modifies GPNMB secretion, we stimulated BMDMs from WT and G2019S LRRK2 knock-in mice with bafilomycin A1 in the absence or presence of the LRRK2 kinase inhibitor MLi-2 (*30*). LRRK2 kinase gain of function resulted in increased GPNMB secretion, as was seen in the CSF of LRRK2 G2019S mutation carriers. Vice versa, LRRK2 kinase inhibition reduced GPNMB secretion, both in WT and G2019S macrophages (Fig. 5C) and in iPSC-derived microglia (Fig. 5D). Consistent with kinase inhibition, we also observed reduced GPNMB secretion in LRRK2 KO RAW264.7 macrophages (Fig. 5E). LRRK2 has been implicated in a multitude of cellular pathways, including in the regulation of TFEB-regulated transcription (*31*). LRRK2 kinase inhibition or the LRRK2 G2019S mutation had no impact on total GPNMB protein levels in BMDMs (Fig. 5, F and G) or iPSC-derived microglia (fig. S6) nor did LRRK2 KO in RAW264.7 cells (Fig. 5, H and I). Western blotting or immunofluorescence imaging for the phosphorylation of the LRRK2 substrate Rab10 confirmed LRRK2 kinase inhibition, overactivation, or loss of function (Fig. 5, F to I and fig. S6), indicating that LRRK2-mediated alterations in TFEB activity are unlikely to underlie alterations in GPNMB secretion.

### LRRK2 modulates GPNMB lysosomal recruitment and lysosomal exocytosis

**Fig. 6.**
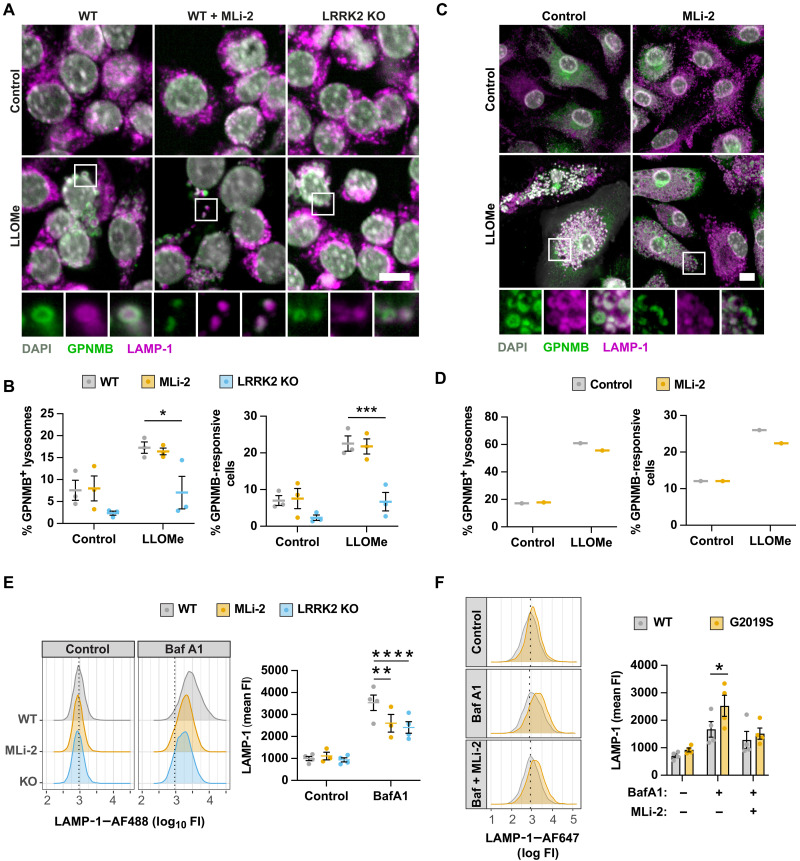
LRRK2 modulates GPNMB lysosomal recruitment and lysosomal exocytosis. (**A**) WT and LRRK2 KO RAW264.7 macrophages were pretreated with MLi-2 (100 nM) and stimulated with LLOMe (1 mM) for 1 hour. GPNMB-LAMP-1 colocalization was assessed by high-content imaging. Scale bar, 10 μm. (**B**) Quantification of the percentage of GPNMB-positive lysosomes and the percentage of GPNMB-responsive cells from (A). Mean ± SEM, *n* = 3. (**C**) WT BMDMs were pretreated with MLi-2 (100 nM) and stimulated with LLOMe (1 mM) for 1 hour. GPNMB–LAMP-1 colocalization was assessed by high-content imaging. Scale bar, 10 μm. (**D**) Quantification of the percentage of GPNMB-positive lysosomes and the percentage of GPNMB-responsive cells from (C). *n* = 1. (**E**) WT and LRRK2 KO RAW264.7 macrophages were pretreated with MLi-2 (100 nM) and stimulated with bafilomycin A1 (100 nM) for 4 hours. Lysosomal exocytosis was estimated by measuring LAMP-1 on the cell surface by flow cytometry. Graphs show mean ± SEM, *n* = 3 to 4. (**F**) WT and LRRK2 G2019S BMDMs were pretreated with MLi-2 (100 nM) and stimulated with bafilomycin A1 (100 nM) for 6 hours. Lysosomal exocytosis was estimated by measuring LAMP-1 on the cell surface by flow cytometry. Graphs show mean ± SEM, *n* = 4.

**Fig. 7.**
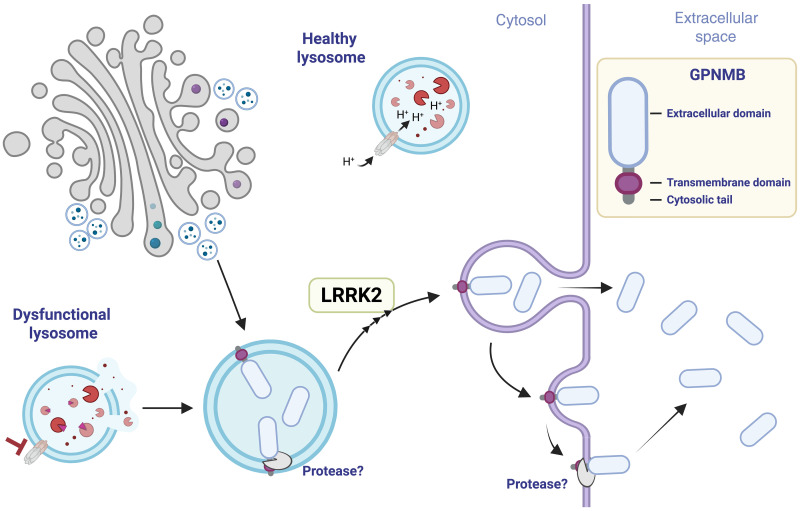
Lysosomal dysfunction results in GPNMB secretion via lysosomal exocytosis. Schematic of the proposed model of GPNMB secretion. GPNMB is not present on healthy lysosomes, but lysosomal dysfunction recruits GPNMB to the lysosomal membrane, most likely from a perinuclear population of endosomes. The dysfunctional lysosomes get exocytosed resulting in the release of the cleaved fragment of GPNMB. GPNMB gets cleaved by proteases, such as ADAM10, either at the lysosome or the plasma membrane. Created in BioRender. Herbst, S. (2025) https://BioRender.com/acpbxru.

To gain an insight into how LRRK2 modulates GPNMB secretion, we first confirmed that GPNMB lysosomal recruitment was maintained in the absence of LRRK2 kinase activity. MLi-2 treatment had a minimal impact on GPNMB recruitment in RAW264.7 macrophages (Fig. 6, A and B) and in BMDMs (Fig. 6, C and D). However, LRRK2 KO resulted in a strong reduction in GPNMB recruitment and a concomitant reduction in the overall number of cells that showed GPNMB lysosomal recruitment (Fig. 6, A and B), indicating that a complete loss of LRRK2 might alter the lysosomal stress response. As LRRK2 has been implicated in lysosomal secretion (*14*, *32*), we assessed lysosomal secretion by measuring the presence of LAMP-1 on the plasma membrane. LRRK2 KO and LRRK2 kinase inhibition strongly reduced lysosomal exocytosis in RAW264.7 cells (Fig. 6E). Vice versa, lysosomal exocytosis was increased in G2019S macrophages, which could be reversed by LRRK2 kinase inhibition (Fig. 6F). These results indicate that LRRK2 modulates GPNMB secretion predominantly by modulating lysosomal exocytosis. In summary, we propose that dysfunctional lysosomes are exocytosed, resulting in the secretion of the GPNMB extracellular domain, a process which is enhanced in the presence of LRRK2 gain-of-function mutations (Fig. 7). Therefore, we propose GPNMB as a secreted biomarker for lysosomal dysfunction.

## DISCUSSION

Because of the association of elevated secreted GPNMB levels to PD risk, GPNMB has emerged as a candidate biomarker and potential therapeutic target in PD. This study identifies lysosomal dysfunction as a trigger of GPNMB secretion and highlights LRRK2 as a modulator of this.

GPNMB is functionally associated with a disease-associated microglia phenotype, which was initially described in mouse models of Alzheimer’s disease, but similar transcriptional signatures have been validated in human tissue across a multitude of neurodegenerative diseases including PD (*33*, *34*). Although Smajić *et al.* (*8*) described GPNMB as a marker for proinflammatory microglia in iPD, we also noted that GPNMB expressing microglia display a transcriptional signature of lipid dyshomeostasis and a high degree of expression of lysosome-related transcripts, including the MiT family transcription factor MiTF, suggesting an ongoing high demand on lysosomal function in these microglia.

Our data indicate that as well as being responsive to lysosomal stress on a transcriptional level, GPNMB secretion is triggered by lysosomal dysfunction. Lysosomal dysfunction results in recruitment of GPNMB to the lysosomal membrane and secretion via lysosomal exocytosis. Although predominantly known for their degradative ability and as a signaling hub (*22*), lysosomes have gained recognition as a secretory compartment. Secreted lysosomes tend to be characterized by a less acidic luminal pH and impaired degrative ability (*20*), implying that lysosomal dysfunction precedes secretion. Coupled with the finding that GPNMB is only present on lysosomes under stress conditions, it is intriguing to speculate that GPNMB secretion represents a “danger” signal, signaling lysosomal stress in macrophages to surrounding cells.

This study uses pharmacological means to induce lysosomal stress. However, α-synuclein aggregates and tau fibrils have been shown to deacidify the lysosomal lumen and cause lysosomal damage akin to the perturbation used in this study (*35*–*37*). Further to this, α-synuclein has been shown to be released via lysosomal exocytosis (*21*, *38*), making it likely that microglia in the Parkinson’s brain experience similar stress.

The signaling events that follow GPNMB secretion remain largely uncharacterized. Secreted GPNMB is reported to have not only anti-inflammatory but also senescence-inducing properties and is thought to signal via CD44 (*39*–*41*). In the context of PD, GPNMB has been highlighted as a receptor for α-synuclein uptake (*3*). Whether secreted GPNMB can therefore act as an α-synuclein–binding partner requires further investigation. Our findings also highlight an interplay between GPNMB secretion and the Parkinson’s risk gene *LRRK2*. The LRRK2 G2019S gain-of-function mutations increased GPNMB secretion, whereas inhibition or loss of LRRK2 kinase function decreased GPNMB secretion substantially. Of note, high GPNMB levels have also been observed in the CSF of carriers of an LRRK2 risk variant that increases PD risk independent of the LRRK2 G2019S mutation (*42*, *43*), further strengthening the connection between LRRK2 and GPNMB release.

On a functional level, LRRK2 can be linked to various secretory events by way of its regulation of Rab proteins and therefore vesicular trafficking (*40*). It is of interest to note that LRRK2 is highly expressed in alveolar type 2 cells, which harbor specialized secretory lysosomes. Abnormal morphology of these organelles was observed early on in LRRK2 KO mice, and a potential impact on lung function remains a concern for the use of LRRK2 kinase inhibitors in clinical practice (*30*, *44*, *45*). LRRK2 has been shown to mediate lysosomal secretion in response to luminal alkalinization (*14*) and is recruited to lysosomes in response to lysosomal stress via unconventional autophagy (*32*, *46*). These data point to LRRK2 being recruited to a subset of lysosomes, which might be characterized by a less catalytic environment. This idea is strengthened by the finding that we did not observe a general presence of lysosomal catalytic proteins in the CSF of LRRK2 mutation carriers; however, whether GPNMB has a unique standing as a lysosomal secreted protein needs further investigation.

LRRK2 phosphorylates a subset of Rab guanosine triphosphatases (*47*), including Rab10, which is directly involved in lysosomal exocytosis (*48*), providing a potential mechanism for LRRK2 modulating GPNMB secretion. However, at this point, we cannot exclude that the altered secretion is due to LRRK2 directly affecting lysosomal properties such as acidification or the lysosomal stress response (*12*, *49*), which in turn also could affect the degree of lysosomal exocytosis. As elevated GPNMB CSF levels alone increase PD risk, these findings also raise the possibility that GPNMB may contribute to heightened PD risk in LRRK2 mutation carriers.

Together, this study highlights GPNMB as a secreted biomarker for lysosomal stress. Although the increased GPNMB levels in iPD indicate that lysosomal dysfunction is a common feature in PD, we postulate that GPNMB will act as a macrophage lysosomal stress marker in a wide spectrum of diseases. The link to LRRK2 biology is intriguing and further highlights the need to understand the downstream consequences of GPNMB secretion to fully understand its potential as a biomarker and putative drug target for PD.

## MATERIALS AND METHODS

### Cell culture

Murine BMDMs were generated by differentiating bone marrow from WT and LRRK2 G2019S knock-in mice (provided by D. Alessi, University of Dundee) in murine granulocyte-macrophage colony-stimulating factor (50 ng/ml; 576306, BioLegend) in Dulbecco’s modified Eagle’s medium (DMEM)/10% fetal calf serum (FCS) for 7 days. RAW264.7 WT [American Type Culture Collection (ATCC), catalog no. SC-6003, RRID:CVCL_UL71) and LRRK2 KO macrophages (ATCC, catalog no. SC-4, RRID:CVCL_UL72) were obtained from ATCC and cultured in DMEM/10% FCS. HEK293T cells were obtained from ATCC (ATCC CRL-3216; RRID:CVCL_0063) and cultured in DMEM/10% FCS. If HEK293T cells were seeded on glass coverslips, then the coverslips were pretreated with poly-d-lysine (Gibco, Thermo Fisher Scientific). Detailed tissue culture protocols can be found under dx.doi.org/10.17504/protocols.io.

The human iPSC line KOLF2.1J were obtained from JAX (#JIPSC001000; RRID:CVCL_B5P3) and differentiated into microglia following a primitive hematopoiesis protocol (*50*). Myeloid embryoid bodies (MEBs) were generated by culturing 15,000 cells per well in StemFlex media supplemented with bone morphogenetic protein 4 (50 ng/ml), vascular endothelial growth factor (50 ng/ml), and stem cell factor (20 ng/ml) in ultralow attachment plates. After 4 days, the MEBs were transferred to T-75 flasks and maintained in X-VIVO 15 media containing macrophage colony-stimulating factor (MCSF;100 ng/ml) and interleukin-3 (IL-3; 25 ng/ml). Microglia-like cells were released into the media over 4 weeks, harvested weekly, and further matured in DMEM-F12 base media [DMEM/F-12 + neurobasal medium + N-2 supplement + B27 supplement + GlutaMAX + NEAA+ 2-mercaptoethanol (50 μM)] supplemented with IL-34 (100 ng/ml), MCSF (25 ng/ml), and transforming growth factor–β1 (5 ng/ml). All cells were cultured at 37°C, 5% CO_2_.

### Inhibitors and cell stimulations

Bafilomycin A1 (SML1661, Sigma-Aldrich) was used at 10 nM for enzyme-linked immunosorbent assay (ELISA) sample preparation or 100 nM for short-term treatment. LLOMe (H-Leu-Leu-OMe HBr, #4000725, Bachem Biochemica) was used at 500 μM for ELISA sample preparation or at 1 mM LLOMe for short-term treatments. GW280264 X (#7030, Tocris) was used at 3 μM, GW4869 (#6741, Tocris) was used at 0.3 to 10 μM, ML SA1 (#4746, Tocris) was used at 3 to 10 μM, (1R,2R)-ML-SI3 (#HY-134819A, MedChemExpress) was used at 30 to 100 μM, nigericin (#tlrl-nig, Invivogen) was used at 5 μM, the proteasome inhibitor MG-132 (#tlrl-mg132, Invivogen) was used at 5 μM, and LPS (#L3129, Sigma-Aldrich) was used at 10 ng/ml. The LRRK2 kinase inhibitor MLi-2 (#5756, Tocris) was used at 100 nM.

### Plasmids and site-directed mutagenesis

The open reading frame of full-length human GPNMB (NM_001005340.2) cloned into pcDNA3.1-C-EGFP was purchased from GenScript. GPNMB mutant constructs were generated by site-directed mutagenesis using the Q5 SDM kit from New England Biolabs (#E0552S). All plasmids were verified by sequencing. All DNA constructs were maintained in *Escherichia coli* DH5α (#11583117, Thermo Fisher Scientific) and extracted using a plasmid miniprep kit from QIAGEN.

### Antibodies

Antibodies used for Western blotting and immunofluorescence were goat anti-human GPNMB (R&D Systems, catalog no. AF2550, RRID:AB_416615), goat anti-mouse GPNMB (AF2330, R&D Systems), mouse anti–human LAMP-1 (DSHB, catalog no. h4a3, RRID:AB_2296838), rabbit anti–mouse LAMP-1 (Abcam, catalog no. ab208943, RRID:AB_2923327), mouse anti-GFP (Thermo Fisher Scientific, catalog no. MA5-15256, RRID:AB_10979281), rabbit anti-ADAM10 (Abcam, catalog no. ab124695, RRID:AB_10972023), rabbit anti-ADAM17 (Proteintech, catalog no. 29948-1-AP, RRID:AB_2935490), rabbit anti-LRRK2 (Abcam, catalog no. ab133474, RRID:AB_2713963), rabbit anti LRRK2 pS935 (Abcam, catalog no. ab133450, RRID:AB_2732035), rabbit anti-Rab10 pT73 (Abcam, catalog no. ab241060, RRID:AB_2884876), rabbit anti-Rab10 (Abcam, catalog no. ab237703, RRID:AB_2884879), and mouse anti-β-actin (Sigma-Aldrich, catalog no. A1978, RRID:AB_476692). Rat anti-mouse-LAMP-1–AF647 (BioLegend, catalog no. 121610, RRID:AB_571990) was used for fluorescence-activated cell sorting (FACS).

### Immunofluorescence microscopy

Cells seeded on coverslips were fixed with 4% methanol-free paraformaldehyde (PFA; 15710, Electron Microscopy Sciences) in phosphate-buffered saline (PBS) for 15 min at 4°C. The samples were permeabilized and blocked with 0.3% Triton X-100, 5% FCS in PBS for 20 min. Primary antibodies were diluted in PBS containing 5% FCS and incubated for 1 hour at room temperature. The samples were washed three times in PBS and incubated with the secondary antibody diluted in 5% FCS in PBS (anti-goat, anti-mouse, or anti–rabbit–Alexa Fluor 488, Alexa Fluor 568, or Alexa Fluor 647, Invitrogen) for 45 min at room temperature. After three more washes with PBS, nuclear staining was performed using 300 nM 4′,6-diamidino-2-phenylindole (Life Technologies, D3571) in PBS for 10 min. One final wash with PBS was performed before mounting the coverslips on glass slides using Dako mounting medium (Dako Cytomation, S3023). Images were acquired on a Leica SP8 inverted microscope or an OPERA Phenix high-content imaging system. Images acquired on a confocal microscope were analyzed using Fiji (*51*). The JaCOP plugin (*52*) was used to calculate the Mander’s coefficient to estimate the overlap of GPNMB with LAMP-1. Images acquired on the OPERA Phenix were analyzed using Harmony. All representative images were arranged using FigureJ (*53*). A detailed staining protocol can be found under http://dx.doi.org/10.17504/protocols.io.4r3l22yz4l1y/v1.

### GPNMB RT-PCR

RNA was extracted using the Monarch total RNA purification kit from NEB (#T2010S) and converted into cDNA using the LunaScript RT SuperMix (NEB, #M3010L) according to the manufacturer’s instructions. Reverse transcription polymerase chain reaction (RT-PCR) was performed on 10 ng of cDNA using TaqMan Universal PCR Master Mix (#4364340) and TaqMan probes for murine GPNMB (assay ID: Mm01328587_m1) and glyceraldehyde-3-phosphate dehydrogenase (GAPDH; assay ID: Mm99999915_g1), all purchased from Thermo Fisher Scientific. The RT-PCRs were run on a QuantStudio 5 Real-Time PCR machine (Thermo Fisher Scientific) using the following cycling conditions: uracil-DNA glycosylase incubation at 50°C for 2 min, polymerase activation at 95°C for 10 min, followed by 40 cycles of denaturation (95°C for 15 s) and annealing/extension (60°C for 1 min). The data were normalized to GAPDH, and the fold change over the untreated control was calculated using the ΔΔCt method.

### GPNMB ELISA

Supernatants for ELISA were prepared by incubating cells with stimuli for ~18 hours. ELISAs for human GPNMB (DY2550, R&D Systems) and mouse GPNMB (DY2330, R&D Systems) were conducted according to the manufacturer’s instructions. GPNMB release was controlled for total cell numbers per well by staining nuclei directly after sample harvest with Hoechst for 15 min before imaging on a Tecan Spark plate reader. In the case of overexpressed GPNMB, EGFP fluorescence was measured in addition to Hoechst to confirm equal transfection efficiency across constructs.

### Western blotting

Cells were washed once with PBS and lysed in a 1% Triton X/tris-HCl lysis buffer (9803S, Cell Signaling) containing protease and phosphatase inhibitors (#78440, Thermo Fisher Scientific). The samples were denatured at 80°C for 8 min in LDS sample buffer and reducing agent (NuPAGE, Life Technologies) and run on a NuPAGE 4 to 12% bis-tris gel (Life Technologies). The gels were transferred onto a polyvinylidene difluoride membrane using the TurboBlot transfer system (Bio-Rad). The membranes were blocked in 5% semiskinned milk in TBS-T (TBS, 0.1% Tween 20) and incubated with primary antibodies in 5% semiskinned milk in TBS-T at 4°C overnight, followed by incubation with the secondary antibodies in 5% skimmed milk in TBS-T for 1 hour at room temperature. Western blots were imaged using the iBright imaging system (Thermo Fisher Scientific) and quantified by densitometry using Fiji (RRID:SCR_002285; http://fiji.sc) (*51*). A detailed protocol can be found under http://dx.doi.org/10.17504/protocols.io.4r3l22yz4l1y/v1.

### FACS analysis of lysosomal exocytosis

Cells were treated with bafilomycin A1 at 100 nM for 4 to 6 hours and harvested in PBS. Fc receptor binding was blocked using a CD16/CD32 targeting antibody (BD Biosciences, catalog no. 553142, RRID:AB_394657) diluted in 5% FCS/PBS for 10 min on ice. The LAMP-1–AF647 antibody was added at 0.5 μl per sample for 30 min on ice, followed by dead/live stain (Fixable Viability Dye eFluor-450, #65-0863-18, eBioscience) for 10 min on ice. The samples were fixed in 2% PFA/PBS and acquired on a BD FACSCanto flow cytometer. Data were analyzed and plotted using the CytoExploreR package for R (*54*). A detailed protocol for staining can be found under DOI: dx.doi.org/10.17504/protocols.io.n92ld8oyxv5b/v1.

### PPMI SomaScan data analysis

A CSF SomaScan dataset was accessed via the PPMI under project ID 151. The dataset was filtered to only contain baseline data and remove participants labeled as prodromal. Patients with PD were further subcategorized into “GBA1 PD” and “LRRK2 PD.” GBA1 PD consists of carriers of *GBA1* E326K, N370S, T369M, or L444P variants, the IVS2 + 1G>A splice donor variant, and the L29Afs*18 loss-of-function variant, thereby including *GBA1* variants that are characterized as either mild or severe in their impact as an underlying cause for PD (*55*). LRRK2 PD includes LRRK2 G2019S mutation carriers only. LRRK2 mutation carriers that also carry *GBA1* variants were removed from the dataset. As a result, we analyzed 1111 participants (44.5% female, 55.5% male) for GPNMB extracellular domain CSF levels (SomaScan testname = “5080-131_3”). Statistical significance between groups was determined by a Kruskal-Wallis test, followed by paired comparisons against the control group using a Wilcoxon test. All data were processed and analyzed using R [version 4.3.1 (16 June 2023)] (*56*).

### Statistical analysis

Unless otherwise stated, statistical analysis was carried out using GraphPad Prism software. Multiple comparisons were calculated by two-way analysis of variance (ANOVA), followed by Dunnett’s multiple comparisons test to test for differences to control or followed by Sidak multiple comparisons test to test for differences between all groups. *P < 0.05, **P < 0.01, ***P < 0.001, and ****P < 0.0001.

## Supplementary Materials

### This PDF file includes:

Supplementary Materials and Methods Figs. S1 to S6 References
