# Supplementary material for: GPNMB is a biomarker for lysosomal dysfunction and is secreted via LRRK2-modulated lysosomal exocytosis
Source: Sci Adv. 2025 Dec 17;11(51):eadv1434. doi: 10.1126/sciadv.adv1434 (PMC13142706; doi:10.1126/sciadv.adv1434)
Supplement: Supplementary file 1 — Supplementary Materials and Methods Figs. S1 to S6 References [file sciadv.adv1434_sm.pdf]

Science Advances
AAAS

Supplementary Materials for

GPNMB is a biomarker for lysosomal dysfunction and is secreted via LRRK2-modulated lysosomal exocytosis

Erin C. Bogacki *et al.*

Corresponding author: Susanne Herbst, sherbst@rvc.ac.uk

Sci. Adv. **11**, eadv1434 (2025)
DOI: 10.1126/sciadv.adv1434

This PDF file includes:

Supplementary Materials and Methods
Figs. S1 to S6
References

Supplementary materials and methods:

Generation of tetracycline-inducible GPNMB RAW264.7 cells

Murine GPNMB (NM_053110.4) was cloned from pcDNA3.1-mGPNMB-EGFP into pSLIK-Neo *(57)* via Gateway recombination and packaged into Lentiviral particles in HEK293 cells using a third-generation Lentiviral packaging system *(58)*. RAW264.7 GPNMB KO macrophages, kindly provided by Jonathan D. Smith *(59)*, were infected with the Lentiviral particles and selected with Geneticin (#10131035, ThermoFisher Scientific) for two weeks. To confirm tetracycline-inducible GPNMB expression, the cells were treated with increasing concentrations of doxycycline (#D5207, Sigma-Aldrich) for 24-48 hrs, and GPNMB expression was assessed by Western Blotting as outlined in the main materials and methods section.

Plasmids

**Plasmid**	**Reference**
pcDNA3.1-mGPNMB-EGFP	This study (synthesized by Genscript)
pSLIK-Neo	Addgene 25735
pSLIK-Neo-mGPNMB	This study
pMDLg/pRRE	Addgene 12251
pVSV-G	Addgene 12259
pRSV-Rev	Addgene 12253

Inhibitors and cell treatments

HEK293 cells were treated with Nocodazole (# HY-13520, MedChemExpress) at 10 µM for 2hrs to destabilise microtubules.
RAW264.6 cells were treated with doxycycline (#D5207, Sigma-Aldrich) from 62.5 – 1000 ng/ml for 48 hrs. To measure GPNMB secretion, Bafilomycin A1 (SML1661, Sigma-Aldrich) was added at 10 nM after 24 hrs of doxycycline treatment without removing the doxycycline.

Additional Antibodies used for ICC

Primary antibodies used for immunofluorescence were mouse-anti-Rab7 (#95746, Cell Signaling Technology; RRID:AB_2800252), mouse-anti-EEA1 (#48453, Cell Signaling Technology; RRID:AB_2920538), rabbit-anti-TGN46 (Proteintech Cat# 13573-1-AP, RRID:AB_10597396) and rabbit-anti-GM130 (#12480, Cell Signaling Technology; RRID:AB_2797933).

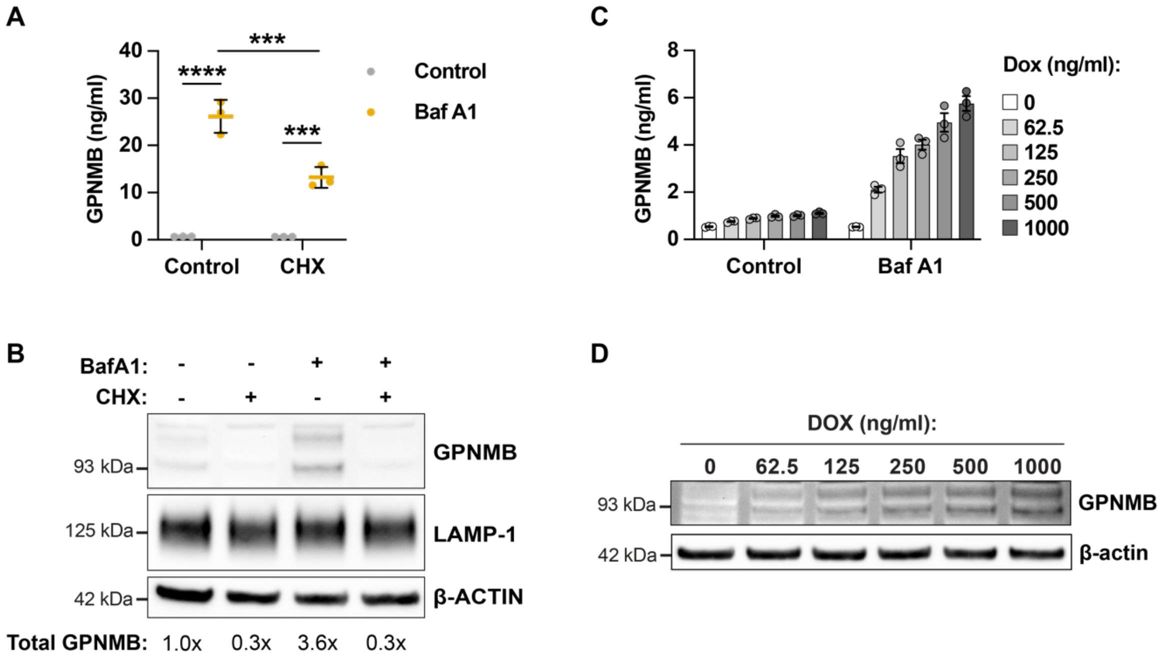

Fig. S1: GPNMB transcription and secretion are uncoupled. **(A-B)** RAW264.7 macrophages were treated with 1 µg/ml Cycloheximide and 10 nM Bafilomycin A1 overnight. **(A)** GPNMB secretion was measured by ELISA. Mean +/- SEM, n=3. **(B)** GPNMB and LAMP-1 levels were assessed by Western Blotting. The numbers underneath the blot indicate total GPNMB levels (precursor + mature form), normalised to untreated control. **(C-D)** RAW264.7 macrophages expressing murine GPNMB from a tetracycline-inducible promoter were treated with the indicated concentrations of Doxycycline (Dox) for 24 hrs, followed by stimulation with 10 nM Bafilomycin A1 overnight. **(C)** GPNMB secretion was measured by ELISA. Mean +/- SEM, n=3. **(D)** GPNMB levels were assessed by Western Blotting after 24 hrs of doxycycline treatment.

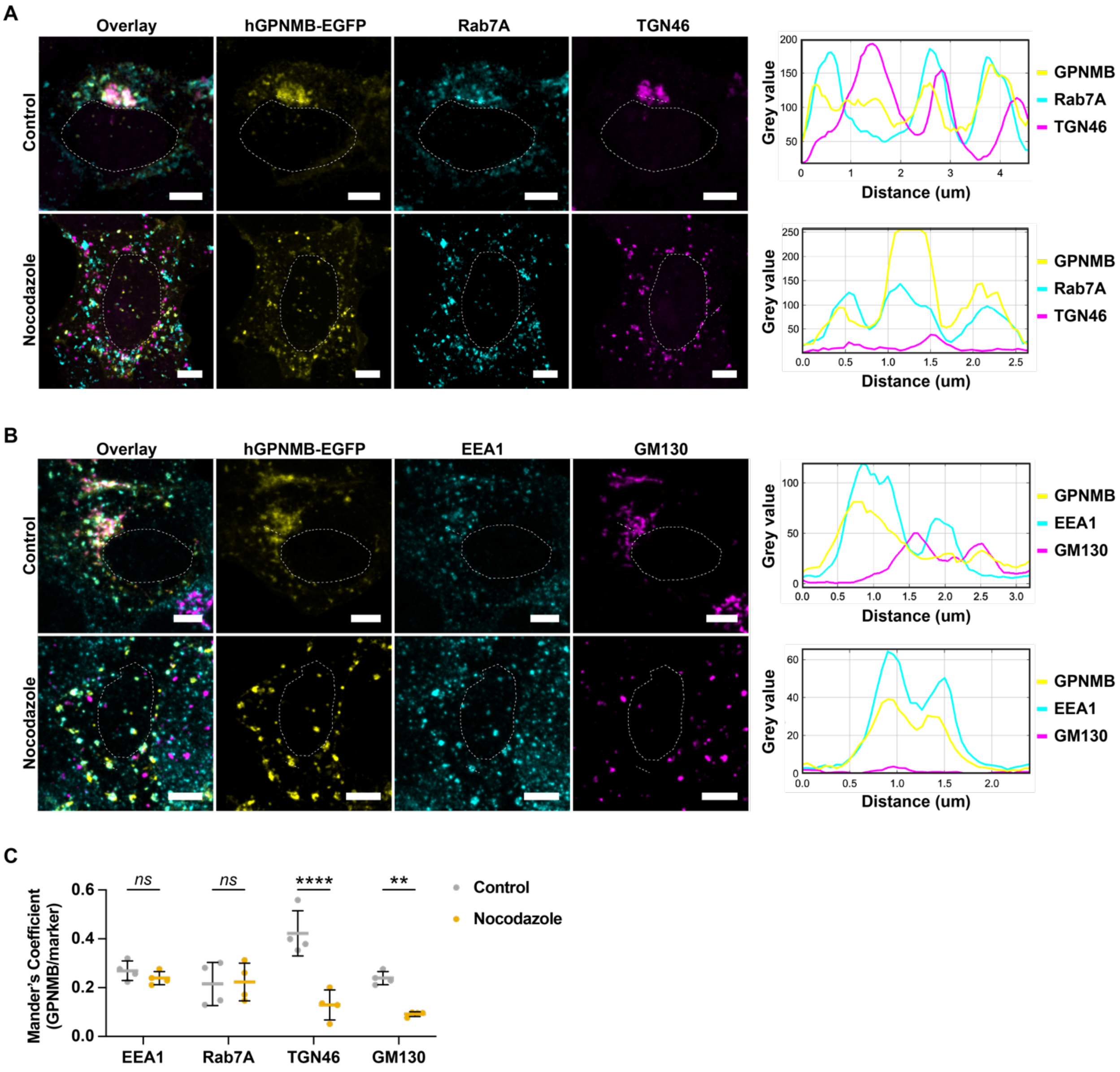

Fig. S2: Localisation of overexpressed GPNMB in HEK293 cells. HEK293 cells were transfected with pcDNA3.1-hGPNMB-EGFP and treated with Nocodazole (10 µM) for 2 hrs where indicated. Cells were stained for **(A)** Rab7A and TGN46 or **(B)** EEA1 and GM130 to assess endosomal, trans-Golgi and Golgi localisation. The nuclear outline is shown, as is a dotted line along which a grey value profile plot for the indicated markers was generated. Scale bar = 5 µm. **(C)** The fraction of GPNMB overlapping with the respective marker was calculated by Mander's Coefficient using the JaCOP plugin in ImageJ. Mean +/- SEM from two independent experiments with two technical replicates.

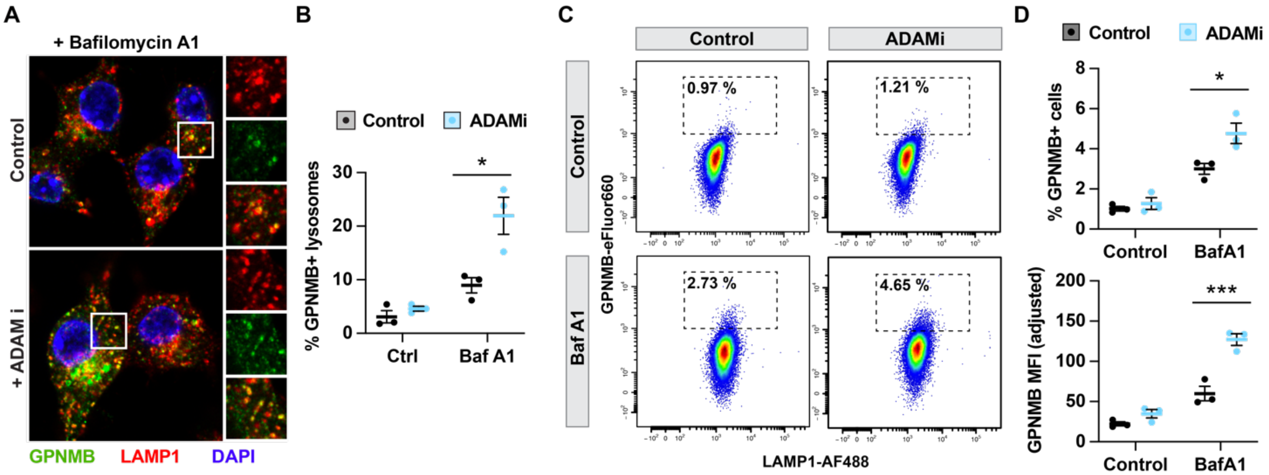

Fig. S3: ADAM10/17 inhibition increases GPNMB lysosomal and cell surface localisation. RAW264.7 macrophages were pretreated with 3 μM ADAM10/17 inhibitor, followed by a 4 hr stimulation with 100 nM Bafilomycin A1. **(A)** GPNMB co-localisation with the lysosomal marker LAMP-1 was assessed by high-content imaging. **(B)** Quantification of (A) showing mean % of GPNMB+ lysosomes +/- SEM of three independent experiments. **(C)** GPNMB cell surface localisation was assessed by flow cytometry. **(D)** Quantification of (C) showing mean +/- SEM of three independent experiments.

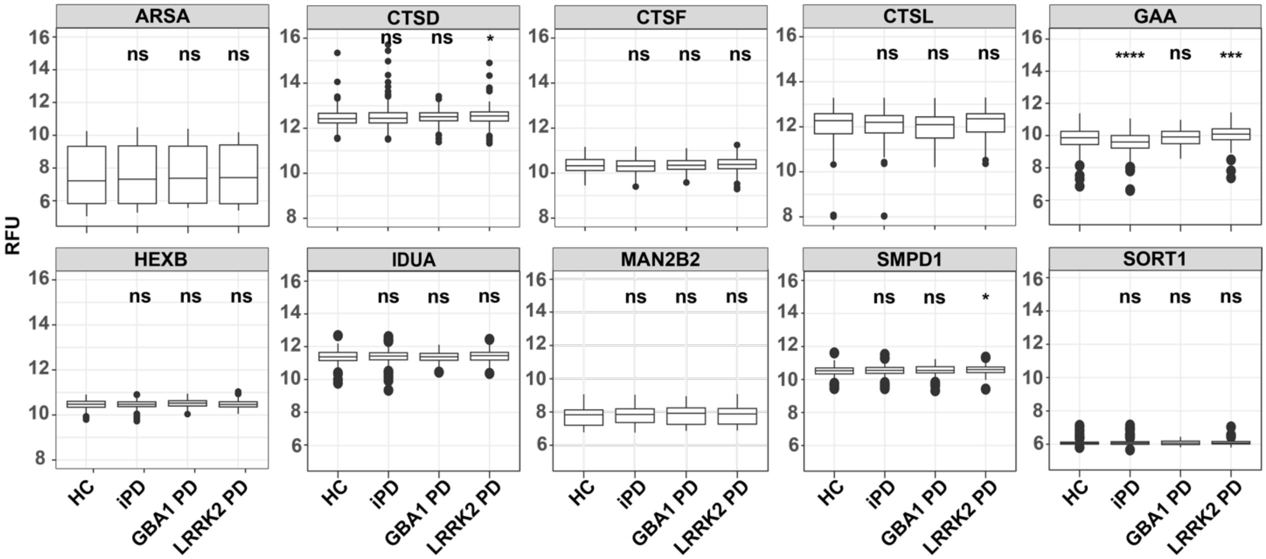

Fig. S4: The impact of LRRK2 on the secreted lysosomal CSF proteome. SomaScan data of lysosomal luminal proteins in the CSF of healthy controls (HC), idiopathic PD patients (iPD), GBA1 PD patients and LRRK2 G2019S PD patients do not indicate that LRRK2 G2019S leads to general secretion of lysosomal content.

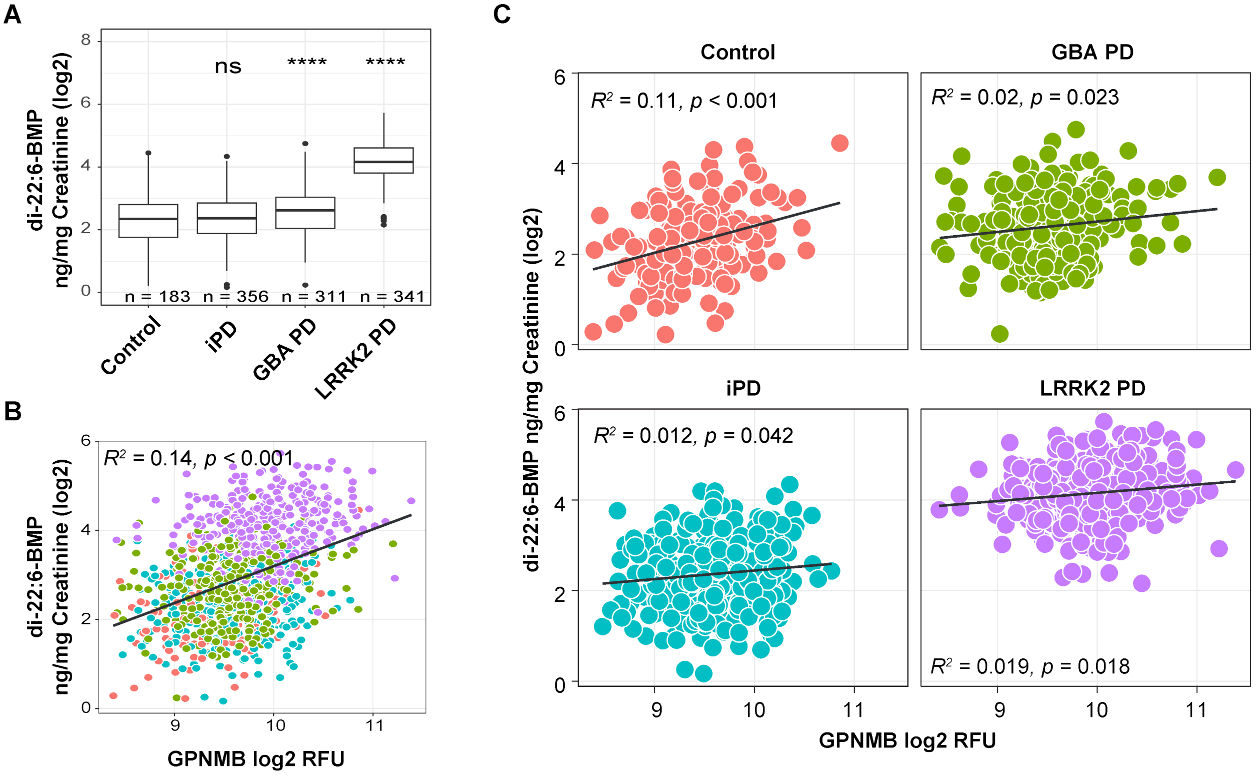

Fig. S5: GPNMB CSF and di-22:5-BMP urine levels do not correlate. (A) Urine di-22:6-BMP levels in healthy controls (HC), idiopathic PD patients (iPD), GBA1 PD patients and LRRK2 G2019S PD patients indicate higher di-22:5-BMP levels in LRRK2 mutation carriers. **(B)** Correlation of GPNMB CSF levels and di-22:5-BMP urine levels. **(C)** Same data as (B) split by healthy controls, iPD, GBA PD and LRRK2 PD. The colours in (B) and (C) correspond to each other.

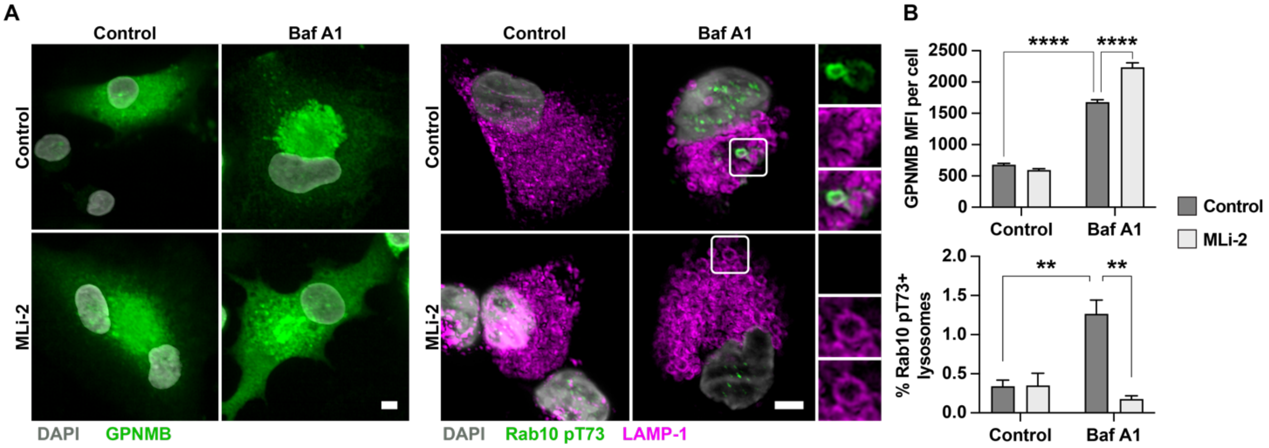

Fig. S6: Controls for Figure 5. iPSC-derived microglia were pretreated with 100 nM MLi-2, followed by stimulation with 10 nM Bafilomycin A1 overnight. **(A)** GPNMB protein abundance and Rab10 pT73 lysosomal co-localisation was assessed by high-content imaging. **(B)** Quantification of (A) showing GPNMB mean fluorescence intensity (MFI) per cell and the % of Rab10 pT73+ lysosomes. Graphs show the mean +/- SEM of ~4000 cells per condition, combined from two independent experiments. ***$p<0.01$*, ****$p<000.1$; Two-way ANOVA with Sidak's multiple comparisons post-test.

REFERENCES

1. M. A. Nalls, C. Blauwendraat, C. L. Vallerga, K. Heilbron, S. Bandres-Ciga, D. Chang, M. Tan, D. A. Kia, A. J. Noyce, A. Xue, J. Bras, E. Young, R. von Coelln, J. Simón-Sánchez, C. Schulte, M. Sharma, L. Krohn, L. Pihlstrom, A. Siitonen, H. Iwaki, H. Leonard, F. Faghri, J. R. Gibbs, D. G. Hernandez, S. W. Scholz, J. A. Botia, M. Martinez, J.-C. Corvol, S. Lesage, J. Jankovic, L. M. Shulman, M. Sutherland, P. Tienari, K. Majamaa, M. Toft, O. A. Andreassen, T. Bangale, A. Brice, J. Yang, Z. Gan-Or, T. Gasser, P. Heutink, J. M. Shulman, N. Wood, D. A. Hinds, J. A. Hardy, H. R. Morris, J. Gratten, P. M. Visscher, R. R. Graham, A. B. Singleton, 23andMe Research Team, System Genomics of Parkinson's Disease (SGPD) Consortium and the International Parkinson's Disease Genomics Consortium, Identification of novel risk loci, causal insights, and heritable risk for Parkinson's disease: A meta-analysis of genome-wide association studies. *Lancet Neurol.* **18**, 1091–1102 (2019).

2. M. N. Murthy, UKBEC, C. Blauwendraat, S. Guelfi, J. Hardy, P. A. Lewis, D. Trabzuni, IPDGC, Increased brain expression of GPNMB is associated with genome wide significant risk for Parkinson's disease on chromosome 7p15.3. [Preprint] (2017). https://doi.org/10.1007/s10048-017-0514-8.

3. M. E. Diaz-Ortiz, Y. Seo, M. Posavi, M. C. Cordon, E. Clark, N. Jain, R. Charan, M. D. Gallagher, T. L. Unger, N. Amari, R. T. Skrinak, R. Davila-Rivera, E. M. Brody, N. Han, R. Zack, V. M. Van Deerlin, T. F. Tropea, K. C. Luk, E. B. Lee, D. Weintraub, A. S. Chen-Plotkin, GPNMB confers risk for Parkinson's disease through interaction with α-synuclein. *Science* **377**, eabk0637 (2022).

4. R. Brendza, H. Lin, K. Stark, O. Foreman, J. Tao, A. Pierce, H. Ngu, K. Shen, A. E. Easton, T. Bhangale, D. Chang, B. Bingol, B. A. Friedman, Genetic ablation of Gpnmb does not alter synuclein-related pathology. *Neurobiol. Dis.* **159**, 105494 (2021).

5. C.-F. Yang, S.-P. Lin, C.-P. Chiang, Y.-H. Wu, W. S. H'ng, C.-P. Chang, Y.-T. Chen, J.-Y. Wu, Loss of GPNMB causes autosomal-recessive amyloidosis cutis dyschromica in humans. *Am. J. Hum. Genet.* **102**, 219–232 (2018).

6. W. Qin, H. Wang, W. Zhong, J. Bai, J. Qiao, Z. Lin, Amyloidosis cutis dyschromica cases caused by GPNMB mutations with different inheritance patterns. *J. Dermatol. Sci.* **104**, 48–54 (2021).

7. M. J. C. Van Der Lienden, P. Gaspar, R. Boot, J. M. F. G. Aerts, M. Van Eijk, Glycoprotein non-metastatic protein B: An emerging biomarker for lysosomal dysfunction in macrophages. *Int. J. Mol. Sci.* **20**, 66 (2018).

8. S. Smajić, C. A. Prada-Medina, Z. Landoulsi, J. Ghelfi, S. Delcambre, C. Dietrich, J. Jarazo, J. Henck, S. Balachandran, S. Pachchek, C. M. Morris, P. Antony, B. Timmermann, S. Sauer, S. L. Pereira, J. C. Schwamborn, P. May, A. Grünewald, M. Spielmann, Single-cell sequencing of human midbrain reveals glial activation and a Parkinson-specific neuronal state. *Brain* **145**, 964–978 (2022).

9. K. L. Carey, G. L. C. Paulus, L. Wang, D. R. Balce, J. W. Luo, P. Bergman, I. C. Ferder, L. Kong, N. Renaud, S. Singh, M. Kost-Alimova, B. Nyfeler, K. G. Lassen, H. W. Virgin, R. J. Xavier, TFEB transcriptional responses reveal negative feedback by BHLHE40 and BHLHE41. *Cell Rep.* **33**, 108371 (2020).

10. N. Bhore, E. C. Bogacki, B. O'Callaghan, H. Plun-Favreau, P. A. Lewis, S. Herbst, Common genetic risk for Parkinson's disease and dysfunction of the endo-lysosomal system. *Philos. Trans. R. Soc. Lond. B Biol. Sci.* **379**, 20220517 (2024).

11. M. Rizig, S. Bandres-Ciga, M. B. Makarious, O. O. Ojo, P. W. Crea, O. V. Abiodun, K. S. Levine, S. A. Abubakar, C. O. Achoru, D. Vitale, O. A. Adeniji, O. P. Agabi, M. J. Koretsky, U. Agulanna, D. A. Hall, R. O. Akinyemi, T. Xie, M. W. Ali, E. A. Shamim, I. Ani-Osheku, M. Padmanaban, O. M. Arigbodi, D. G. Standaert, A. H. Bello, M. N. Dean, C. O. Erameh, I. Elsayed, T. H. Farombi, O. Okunoye, M. B. Fawale, K. J. Billingsley, F. A. Imarhiagbe, P. A. Jerez, E. U. Iwuozo, B. Baker, M. A. Komolafe, L. Malik, P. O. Nwani, K. Daida, E. O. Nwazor, A. Miano-Burkhardt, Y. W. Nyandaiti, Z.-H. Fang, Y. O. Obiabo, J. H. Kluss, O. A. Odeniyi, D. G. Hernandez, F. E. Odiase, N. Tayebi, F. I. Ojini, E. Sidranksy, G. A. Onwuegbuzie, A. M. D'Souza, G. O. Osaigbovo, B. Berhe, N. Osemwegie, X. Reed, O. O. Oshinaike, H. L. Leonard, F. M. Otubogun, C. X. Alvarado, S. I. Oyakhire, S. I. Ozomma, S. C. Samuel, F. T. Taiwo, K. W. Wahab, Y. A. Zubair, H. Iwaki, J. J. Kim, H. R. Morris, J.

Hardy, M. A. Nalls, K. Heilbron, L. Norcliffe-Kaufmann, Nigeria Parkinson Disease Research Network, International Parkinson's Disease Genomics Consortium Africa, Black and African American Connections to Parkinson's Disease Study Group, 23andMe Research Team, C. Blauwendraat, H. Houlden, A. Singleton, N. U. Okubadejo, Global Parkinson's Genetics Program, Identification of genetic risk loci and causal insights associated with Parkinson's disease in African and African admixed populations: A genome-wide association study. *Lancet Neurol.* **22**, 1015–1025 (2023).

12. S. Herbst, P. Campbell, J. Harvey, E. M. Bernard, V. Papayannopoulos, N. W. Wood, H. R. Morris, M. G. Gutierrez, LRRK2 activation controls the repair of damaged endomembranes in macrophages. *EMBO J.* **39**, e104494 (2020).

13. L. Bonet-Ponce, A. Beilina, C. D. Williamson, E. Lindberg, J. H. Kluss, S. Saez-Atienzar, N. Landeck, R. Kumaran, A. Mamais, C. K. E. Bleck, Y. Li, M. R. Cookson, LRRK2 mediates tubulation and vesicle sorting from lysosomes. *Sci. Adv.* **6**, eabb2454 (2020).

14. T. Eguchi, T. Kuwahara, M. Sakurai, T. Komori, T. Fujimoto, G. Ito, S.-I. Yoshimura, A. Harada, M. Fukuda, M. Koike, T. Iwatsubo, LRRK2 and its substrate Rab GTPases are sequentially targeted onto stressed lysosomes and maintain their homeostasis. *Proc. Natl. Acad. Sci. U.S.A.* **115**, E9115–E9124 (2018).

15. M.-J. Dolan, M. Therrien, S. Jereb, T. Kamath, V. Gazestani, T. Atkeson, S. E. Marsh, A. Goeva, N. M. Lojek, S. Murphy, C. M. White, J. Joung, B. Liu, F. Limone, K. Eggan, N. Hacohen, B. E. Bernstein, C. K. Glass, V. Leinonen, M. Blurton-Jones, F. Zhang, C. B. Epstein, E. Z. Macosko, B. Stevens, Exposure of iPSC-derived human microglia to brain substrates enables the generation and manipulation of diverse transcriptional states in vitro. *Nat. Immunol.* **24**, 1382–1390 (2023).

16. S. K. Loftus, A. Antonellis, I. Matera, G. Renaud, L. L. Baxter, D. Reid, T. G. Wolfsberg, Y. Chen, C. Wang, NISC Comparative Sequencing Program, M. K. Prasad, S. L. Bessling, A. S. McCallion, E. D. Green, D. C. Bennett, W. J. Pavan, Gpnmb is a melanoblast-expressed, MITF-dependent gene. *Pigment Cell Melanoma Res.* **22**, 99–110 (2009).

17. P. V. Hornbeck, B. Zhang, B. Murray, J. M. Kornhauser, V. Latham, E. Skrzypek, PhosphoSitePlus, 2014: Mutations, PTMs and recalibrations. *Nucleic Acids Res.* **43**, D512–D520 (2015).

18. D. J. Owen, B. M. Collins, P. R. Evans, ADAPTORS FOR CLATHRIN COATS: Structure and function. *Annu. Rev. Cell Dev. Biol.* **20**, 153–191 (2004).

19. C. Kienzle, J. von Blume, Secretory cargo sorting at the trans-Golgi network. *Trends Cell Biol.* **24**, 584–593 (2014).

20. E. Néel, M. Chiritoiu-Butnaru, W. Fargues, M. Denus, M. Colladant, A. Filaquier, S. E. Stewart, S. Lehmann, C. Zurzolo, D. C. Rubinsztein, P. Marin, M.-L. Parmentier, J. Villeneuve, The endolysosomal system in conventional and unconventional protein secretion. *J. Cell Biol.* **223**, e202404152 (2024).

21. T. Tsunemi, T. Perez-Rosello, Y. Ishiguro, A. Yoroisaka, S. Jeon, K. Hamada, M. Rammonhan, Y. C. Wong, Z. Xie, W. Akamatsu, J. R. Mazzulli, D. J. Surmeier, N. Hattori, D. Krainc, Increased lysosomal exocytosis induced by lysosomal ca^{2+} channel agonists protects human dopaminergic neurons from α-synuclein toxicity. *J. Neurosci.* **39**, 5760–5772 (2019).

22. A. Ballabio, J. S. Bonifacino, Lysosomes as dynamic regulators of cell and organismal homeostasis. *Nat. Rev. Mol. Cell Biol.* **21**, 101–118 (2020).

23. H. Furochi, S. Tamura, M. Mameoka, C. Yamada, T. Ogawa, K. Hirasaka, Y. Okumura, T. Imagawa, S. Oguri, K. Ishidoh, K. Kishi, S. Higashiyama, T. Nikawa, Osteoactivin fragments produced by ectodomain shedding induce MMP-3 expression via ERK pathway in mouse NIH-3T3 fibroblasts. *FEBS Lett.* **581**, 5743–5750 (2007).

24. C. Hundhausen, D. Misztela, T. A. Berkhout, N. Broadway, P. Saftig, K. Reiss, D. Hartmann, F. Fahrenholz, R. Postina, V. Matthews, K.-J. Kallen, S. Rose-John, A. Ludwig, The disintegrin-like metalloproteinase ADAM10 is involved in constitutive cleavage of CX3CL1 (fractalkine) and regulates CX3CL1-mediated cell-cell adhesion. *Blood* **102**, 1186–1195 (2003).

25. V. Murugesan, J. Liu, R. Yang, H. Lin, A. Lischuk, G. Pastores, X. Zhang, W.-L. Chuang, P. K. Mistry, Validating glycoprotein non-metastatic melanoma B (gpNMB, osteoactivin), a new biomarker of Gaucher disease. *Blood Cells Mol. Dis.* **68**, 47–53 (2018).

26. E. B. Moloney, A. Moskites, E. J. Ferrari, O. Isacson, P. J. Hallett, The glycoprotein GPNMB is selectively elevated in the substantia nigra of Parkinson's disease patients and increases after lysosomal stress. *Neurobiol. Dis.* **120**, 1–11 (2018).

27. C. Yang, F. H. G. Farias, L. Ibanez, A. Suhy, B. Sadler, M. V. Fernandez, F. Wang, J. L. Bradley, B. Eiffert, J. A. Bahena, J. P. Budde, Z. Li, U. Dube, Y. J. Sung, K. A. Mihindukulasuriya, J. C. Morris, A. M. Fagan, R. J. Perrin, B. A. Benitez, H. Rhinn, O. Harari, C. Cruchaga, Genomic atlas of the proteome from brain, CSF and plasma prioritizes proteins implicated in neurological disorders. *Nat. Neurosci.* **24**, 1302–1312 (2021).

28. R. G. Langston, A. Beilina, X. Reed, A. Kaganovich, A. B. Singleton, C. Blauwendraat, J. R. Gibbs, M. R. Cookson, Association of a common genetic variant with Parkinson's disease is mediated by microglia. *Sci. Transl. Med.* **14**, eabp8869 (2022).

29. K. M. Merchant, T. Simuni, J. Fedler, C. Caspell-Garcia, M. Brumm, K. N. H. Nudelman, E. Tengstrandt, F. Hsieh, R. N. Alcalay, C. Coffey, L. Chahine, T. Foroud, A. Singleton, D. Weintraub, S. Hutten, T. Sherer, B. Mollenhauer, A. Siderowf, C. Tanner, K. Marek, the Parkinson's Progression Markers Initiative, LRRK2 and GBA1 variant carriers have higher urinary bis(monacylglycerol) phosphate concentrations in PPMI cohorts. *NPJ Parkinsons Dis.* **9**, 30 (2023).

30. M. J. Fell, C. Mirescu, K. Basu, B. Cheewatrakoolpong, D. E. DeMong, J. M. Ellis, L. A. Hyde, Y. Lin, C. G. Markgraf, H. Mei, M. Miller, F. M. Poulet, J. D. Scott, M. D. Smith, Z. Yin, X. Zhou, E. M. Parker, M. E. Kennedy, J. A. Morrow, MLi-2, a potent, selective, and centrally active compound for exploring the therapeutic potential and safety of LRRK2 kinase inhibition. *J. Pharmacol. Exp. Ther.* **355**, 397–409 (2015).

31. N. Yadavalli, S. M. Ferguson, LRRK2 suppresses lysosome degradative activity in macrophages and microglia through MiT-TFE transcription factor inhibition. *Proc. Natl. Acad. Sci. U.S.A.* **120**, e2303789120 (2023).

32. T. Eguchi, M. Sakurai, Y. Wang, C. Saito, G. Yoshii, T. Wileman, N. Mizushima, T. Kuwahara, T. Iwatsubo, The V-ATPase-ATG16L1 axis recruits LRRK2 to facilitate the lysosomal stress response. *J. Cell Biol.* **223**, e202302067 (2024).

33. H. Keren-Shaul, A. Spinrad, A. Weiner, O. Matcovitch-Natan, R. Dvir-Szternfeld, T. K. Ulland, E. David, K. Baruch, D. Lara-Astaiso, B. Toth, S. Itzkovitz, M. Colonna, M. Schwartz, I. Amit, A unique microglia type associated with restricting development of Alzheimer's disease. *Cell* **169**, 1276–1290.e17 (2017).

34. R. C. Paolicelli, A. Sierra, B. Stevens, M.-E. Tremblay, A. Aguzzi, B. Ajami, I. Amit, E. Audinat, I. Bechmann, M. Bennett, F. Bennett, A. Bessis, K. Biber, S. Bilbo, M. Blurton-Jones, E. Boddeke, D. Brites, B. Brône, G. C. Brown, O. Butovsky, M. J. Carson, B. Castellano, M. Colonna, S. A. Cowley, C. Cunningham, D. Davalos, P. L. De Jager, B. de Strooper, A. Denes, B. J. L. Eggen, U. Eyo, E. Galea, S. Garel, F. Ginhoux, C. K. Glass, O. Gokce, D. Gomez-Nicola, B. González, S. Gordon, M. B. Graeber, A. D. Greenhalgh, P. Gressens, M. Greter, D. H. Gutmann, C. Haass, M. T. Heneka, F. L. Heppner, S. Hong, D. A. Hume, S. Jung, H. Kettenmann, J. Kipnis, R. Koyama, G. Lemke, M. Lynch, A. Majewska, M. Malcangio, T. Malm, R. Mancuso, T. Masuda, M. Matteoli, B. W. McColl, V. E. Miron, A. V. Molofsky, M. Monje, E. Mracsko, A. Nadjar, J. J. Neher, U. Neniskyte, H. Neumann, M. Noda, B. Peng, F. Peri, V. H. Perry, P. G. Popovich, C. Pridans, J. Priller, M. Prinz, D. Ragozzino, R. M. Ransohoff, M. W. Salter, A. Schaefer, D. P. Schafer, M. Schwartz, M. Simons, C. J. Smith, W. J. Streit, T. L. Tay, L.-H. Tsai, A. Verkhratsky, R. von Bernhardi, H. Wake, V. Wittamer, S. A. Wolf, L.-J. Wu, T. Wyss-Coray, Microglia states and nomenclature: A field at its crossroads. *Neuron* **110**, 3458–3483 (2022).

35. K. Rose, T. Jepson, S. Shukla, A. Maya-Romero, M. Kampmann, K. Xu, J. H. Hurley, Tau fibrils induce nanoscale membrane damage and nucleate cytosolic tau at lysosomes. *Proc. Natl. Acad. Sci. U.S.A.* **121**, e2315690121 (2024).

36. K. Kakuda, K. Ikenaka, A. Kuma, J. Doi, C. Aguirre, N. Wang, T. Ajiki, C.-J. Choong, Y. Kimura, S. M. M. Badawy, T. Shima, S. Nakamura, K. Baba, S. Nagano, Y. Nagai, T. Yoshimori, H. Mochizuki, Lysophagy protects against propagation of α-synuclein

aggregation through ruptured lysosomal vesicles. *Proc. Natl. Acad. Sci. U.S.A.* **121**, e2312306120 (2024).

37. C. Bussi, J. M. P. Ramos, D. S. Arroyo, J. I. Gallea, P. Ronchi, A. Kolovou, J. M. Wang, O. Florey, M. S. Celej, Y. Schwab, N. T. Ktistakis, P. Iribarren, Alpha-synuclein fibrils recruit TBK1 and OPTN to lysosomal damage sites and induce autophagy in microglial cells. *J. Cell Sci.* **131**, jcs226241 (2018).

38. E.-J. Bae, M. Choi, J. T. Kim, D.-K. Kim, M. K. Jung, C. Kim, T.-K. Kim, J. S. Lee, B. C. Jung, S. J. Shin, K. H. Rhee, S.-J. Lee, TNF-α promotes α-synuclein propagation through stimulation of senescence-associated lysosomal exocytosis. *Exp. Mol. Med.* **54**, 788–800 (2022).

39. B. Nickl, F. Qadri, M. Bader, Anti-inflammatory role of Gpnmb in adipose tissue of mice. *Sci. Rep.* **11**, 19614 (2021).

40. M. L. Neal, A. M. Boyle, K. M. Budge, F. F. Safadi, J. R. Richardson, The glycoprotein GPNMB attenuates astrocyte inflammatory responses through the CD44 receptor. *J. Neuroinflammation* **15**, 73 (2018).

41. M. Suda, I. Shimizu, G. Katsuumi, Y. Yoshida, Y. Hayashi, R. Ikegami, N. Matsumoto, Y. Yoshida, R. Mikawa, A. Katayama, J. Wada, M. Seki, Y. Suzuki, A. Iwama, H. Nakagami, A. Nagasawa, R. Morishita, M. Sugimoto, S. Okuda, M. Tsuchida, K. Ozaki, M. Nakanishi-Matsui, T. Minamino, Senolytic vaccination improves normal and pathological age-related phenotypes and increases lifespan in progeroid mice. *Nat. Aging* **1**, 1117–1126 (2021).

42. B. Phillips, D. Western, L. Wang, J. Timsina, Y. Sun, P. Gorijala, C. Yang, A. Do, N.-P. Nykänen, I. Alvarez, M. Aguilar, P. Pastor, J. C. Morris, S. E. Schindler, A. M. Fagan, R. Puerta, P. García-González, I. de Rojas, M. Marquié, M. Boada, A. Ruiz, J. S. Perlmutter, Dominantly Inherited Alzheimer Network (DIAN) Consortia, L. Ibanez, R. J. Perrin, Y. J. Sung, C. Cruchaga, Proteome wide association studies of LRRK2 variants identify novel causal and druggable proteins for Parkinson's disease. *NPJ Parkinsons Dis.* **9**, 107 (2023).

43. J. Lake, X. Reed, R. G. Langston, M. A. Nalls, Z. Gan-Or, M. R. Cookson, A. B. Singleton, C. Blauwendraat, H. L. Leonard, International Parkinson's Disease Genomics Consortium

(IPDGC), coding and noncoding variation in LRRK2 and Parkinson's disease risk. *Mov. Disord.* **37**, 95–105 (2022).

44. G. K. Miller, S. Kuruvilla, B. Jacob, L. LaFranco-Scheuch, V. Bakthavatchalu, J. Flor, K. Flor, J. Ziegler, C. Reichard, P. Manfre, S. Firner, T. McNutt, D. Quay, S. Bellum, G. Doto, P. J. Ciaccio, K. Pearson, J. Valentine, P. Fuller, M. Fell, T. Tsuchiya, T. Williamson, G. Wollenberg, Effects of LRRK2 inhibitors in nonhuman primates. *Toxicol. Pathol.* **51**, 232–245 (2023).

45. M. C. Herzig, C. Kolly, E. Persohn, D. Theil, T. Schweizer, T. Hafner, C. Stemmelen, T. J. Troxler, P. Schmid, S. Danner, C. R. Schnell, M. Mueller, B. Kinzel, A. Grevot, F. Bolognani, M. Stirn, R. R. Kuhn, K. Kaupmann, P. H. Van der Putten, G. Rovelli, D. R. Shimshek, LRRK2 protein levels are determined by kinase function and are crucial for kidney and lung homeostasis in mice. *Hum. Mol. Genet.* **20**, 4209–4223 (2011).

46. A. Bentley-DeSousa, A. Roczniak-Ferguson, S. M. Ferguson, A STING-CASM-GABARAP pathway activates LRRK2 at lysosomes. *J. Cell Biol.* **224**, e202310150 (2025).

47. M. Steger, F. Tonelli, G. Ito, P. Davies, M. Trost, M. Vetter, S. Wachter, E. Lorentzen, G. Duddy, S. Wilson, M. A. Baptista, B. K. Fiske, M. J. Fell, J. A. Morrow, A. D. Reith, D. R. Alessi, M. Mann, Phosphoproteomics reveals that Parkinson's disease kinase LRRK2 regulates a subset of Rab GTPases. *eLife* **5**, e12813 (2016).

48. M. Encarnação, L. Espada, C. Escrevente, D. Mateus, J. Ramalho, X. Michelet, I. Santarino, V. W. Hsu, M. B. Brenner, D. C. Barral, O. V. Vieira, A Rab3a-dependent complex essential for lysosome positioning and plasma membrane repair. *J. Cell Biol.* **213**, 631–640 (2016).

49. A. Härtlova, S. Herbst, J. Peltier, A. Rodgers, O. Bilkei-Gorzo, A. Fearns, B. D. Dill, H. Lee, R. Flynn, S. A. Cowley, P. Davies, P. A. Lewis, I. G. Ganley, J. Martinez, D. R. Alessi, A. D. Reith, M. Trost, M. G. Gutierrez, LRRK2 is a negative regulator of Mycobacterium tuberculosis phagosome maturation in macrophages. *EMBO J.* **37**, e98694 (2018).

50. P. Garcia-Reitboeck, A. Phillips, T. M. Piers, C. Villegas-Llerena, M. Butler, A. Mallach, C. Rodrigues, C. E. Arber, A. Heslegrave, H. Zetterberg, H. Neumann, S. Neame, H. Houlden, J. Hardy, J. M. Pocock, Human induced pluripotent stem cell-derived microglia-like cells harboring TREM2 missense mutations show specific deficits in phagocytosis. *Cell Rep.* **24**, 2300–2311 (2018).

51. J. Schindelin, I. Arganda-Carreras, E. Frise, V. Kaynig, M. Longair, T. Pietzsch, S. Preibisch, C. Rueden, S. Saalfeld, B. Schmid, J.-Y. Tinevez, D. J. White, V. Hartenstein, K. Eliceiri, P. Tomancak, A. Cardona, Fiji: An open-source platform for biological-image analysis. *Nat. Methods* **9**, 676–682 (2012).

52. S. Bolte, F. P. Cordelières, A guided tour into subcellular colocalization analysis in light microscopy. *J. Microsc.* **224**, 213–232 (2006).

53. J. Mutterer, E. Zinck, Quick-and-clean article figures with FigureJ. *J. Microsc.* **252**, 89–91 (2013).

54. D. Hammill, CytoExploreR: Interactive Analysis of Cytometry Data. [Preprint] (2021). https://github.com/DillonHammill/CytoExploreR.

55. S. C. Parlar, F. P. Grenn, J. J. Kim, C. Baluwendraat, Z. Gan-Or, Classification of GBA1 variants in Parkinson's disease: The GBA1-PD browser. *Mov. Disord.* **38**, 489–495 (2023).

56. R Core Team, "R: A language and environment for statistical computing" (R Foundation for Statistical Computing, 2023); https://R-project.org/.

57. K.-J. Shin, E. A. Wall, J. R. Zavzavadjian, L. A. Santat, J. Liu, J.-I. Hwang, R. Rebres, T. Roach, W. Seaman, M. I. Simon, I. D. C. Fraser, A single lentiviral vector platform for microRNA-based conditional RNA interference and coordinated transgene expression. *Proc. Natl. Acad. Sci. U.S.A.* **103**, 13759–13764 (2006).

58. T. Dull, R. Zufferey, M. Kelly, R. J. Mandel, M. Nguyen, D. Trono, L. Naldini, A third-generation lentivirus vector with a conditional packaging system. *J. Virol.* **72**, 8463–8471 (1998).

59. P. Robinet, B. Ritchey, S. W. Lorkowski, A. M. Alzayed, S. DeGeorgia, E. Schodowski, C. A. Traughber, J. D. Smith, Quantitative trait locus mapping identifies the Gpnmb gene as a modifier of mouse macrophage lysosome function. *Sci. Rep.* **11**, 10249 (2021).
